# The REGγ-proteasome forms a regulatory circuit with IκBɛ and NFκB in experimental colitis

**DOI:** 10.1038/ncomms10761

**Published:** 2016-02-22

**Authors:** Jinjin Xu, Lei Zhou, Lei Ji, Fengyuan Chen, Karen Fortmann, Kun Zhang, Qingwu Liu, Ke Li, Weicang Wang, Hao Wang, Wei Xie, Qingwei Wang, Jiang Liu, Biao Zheng, Pei Zhang, Shixia Huang, Tieliu Shi, Biaohong Zhang, Yongyan Dang, Jiwu Chen, Bert W. O'Malley, Robb E. Moses, Ping Wang, Lei Li, Jianru Xiao, Alexander Hoffmann, Xiaotao Li

**Affiliations:** 1 Shanghai Key Laboratory of Regulatory Biology, Shanghai Key Laboratory of Brain Functional Genomics (Ministry of Education), Institute of Biomedical Sciences, East China Normal University, Shanghai 200241, China; 2 The Fifth Hospital of Shanghai, Fudan University, Shanghai 200240, China; 3 Signaling Systems Laboratory and San Diego Center for Systems Biology, University of California, San Diego, 9500 Gilman Drive, La Jolla, California 92093, USA; 4 Department of Microbiology, Immunology, and Molecular Genetics and Institute for Quantitative and Computational Biosciences, University of California, Los Angeles, California 90025, USA; 5 The Institute of Aging Research, School of Medicine, Hangzhou Normal University, Hangzhou, Zhejiang 310036, China; 6 Department of Pathology, the Second Chengdu Municipal Hospital, Chengdu 610017, China; 7 Department of Molecular and Cellular Biology, The Dan L. Duncan Cancer Center, Baylor College of Medicine. One Baylor Plaza, Houston, Texas 77030, USA; 8 Department of Orthopedic Oncology, Changzheng Hospital, The Second Military Medical University, 415 Fengyang Road, Shanghai 200003, China

## Abstract

Increasing incidence of inflammatory bowel disorders demands a better understanding of the molecular mechanisms underlying its multifactorial aetiology. Here we demonstrate that mice deficient for REGγ, a proteasome activator, show significantly attenuated intestinal inflammation and colitis-associated cancer in dextran sodium sulfate model. Bone marrow transplantation experiments suggest that REGγ's function in non-haematopoietic cells primarily contributes to the phenotype. Elevated expression of REGγ exacerbates local inflammation and promotes a reciprocal regulatory loop with NFκB involving ubiquitin-independent degradation of IκBɛ. Additional deletion of *IκBɛ* restored colitis phenotypes and inflammatory gene expression in *REGγ*-deficient mice. In sum, this study identifies REGγ-mediated control of IκBɛ as a molecular mechanism that contributes to NFκB activation and promotes bowel inflammation and associated tumour formation in response to chronic injury.

## Abstract (web summary)

REGγ is a component of ubiquitin-independent 20S proteasome that targets many regulatory proteins for degradation. Here the authors show that REGγ is induced in DSS colitis and promotes degradation of IκBɛ, and that REGγ-deficient mice have less NFκB activation and are more resistant to the disease.

## Graphical abstract

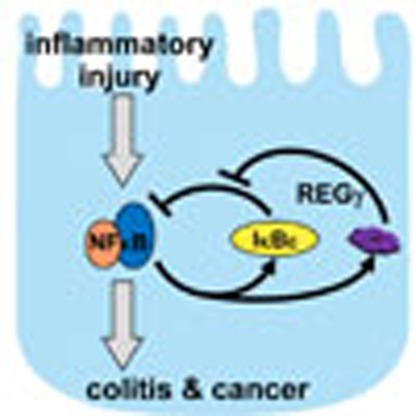

Inflammatory bowel disease (IBD), including ulcerative colitis (UC) and Crohn's disease (CD), is characterized by chronic relapsing intestinal inflammation. IBD is a worldwide health-care problem with significant morbidity. Development of IBD involves a complex interaction between genetic and environmental factors, intestinal microbial flora and immune responses^1^,^2^. Genome-wide searches for IBD susceptibility loci have successfully identified 163 gene loci that contribute to disease susceptibility^3^. Among these genes are the nuclear factor kappa B (NFκB) family members Rel (also known as c-Rel) and Rel A (refs 4, 5), known as key regulators of inflammatory gene expression. Moreover, many pro-inflammatory mediators, such as CXCL5, KC, MIP2, IL-6 and IL-1β are significantly related to IBD. In addition, genes involved in regulation of proteasome functions, such as *USP34*, *WSB1*, *BRE* and the NFκB regulator *TNFAIP3/A20* (refs 4, 6) are among the IBD susceptibility candidates. In the past several decades, advances in the understanding of the molecular pathogenesis in IBD have been made, partly owing to mouse models, which display similar features to UC^1^,^7^,^8^. UC is characterized by diffuse mucosal inflammation limited to the colon. Substantive mucosal ulceration occurs in the colon area with secretion of massive inflammatory mediators and coincident severe inflammation. Moreover, large numbers of neutrophils are often present in the lamina propria and the crypts. In addition, goblet cell mucin is lost. However, many more IBD predisposing factors are yet to be identified, and the underlying molecular mechanisms remain to be characterized. The dextran sodium sulfate (DSS) induced colitis model is an experimental murine model of UC. Although DSS model is not equivalent to human IBD, It has been widely used in the study of bowel inflammation and IBD.

A link between inflammation and cancer has been made for about two millennia^9^,^10^. It is now known that inflammatory diseases increase the risk of developing cancers^11^,^12^. Colon cancer is the third most common cancer in males and the second in females worldwide^13^. Patients with UC or CD are at higher risk for the development of colon cancer^14^. Excessive production of cytokines, chemokines, matrix-degrading enzymes and growth factors in lesions is widely considered as a key factor contributing to tumourigenesis^11^. A combination of DSS and azoxymethane (AOM) serves as a good model system for the study of colitis-associated cancer development (CAC)^15^.

REGγ, also known as PA28γ, 11sγ, PSME3 and Ki antigen, belongs to the 11s family of proteasome activators that bind to and activate 20s core proteins. It degrades a series of target proteins in an ATP- and ubiquitin-independent manner, suggesting a novel regulatory path^16^,^17^,^18^. REGγ is involved in the regulation of a broad range of important physiological processes, including cancer progression^19^, aging^20^, hepatic lipid metabolism^21^ and angiogenesis^22^. REGγ may also play a role in the regulation of innate immunity^23^. However, little is known about its mechanisms in the regulation of inflammatory diseases or its relationship to inflammation-associated cancer. Here we investigate its role in innate immunity and tumour micro-environment.

NFκB signalling plays a pivotal role in inflammatory responses, immune responses, cell growth, tissue differentiation and apoptosis^24^. In resting cells, NFκB is maintained in an inactive, cytoplasmic state in complexes with the IκB family inhibitory proteins. The canonical IκBs comprise IκBα, IκBβ and IκBɛ (ref. 25). Although IκBα is known as the primary regulator of NFκB in response to inflammatory cytokines, no physiological function of IκBɛ outside the haematopoietic compartment has been established^26^,^27^. Two distinct degradation pathways regulate IκB levels, the well-described stimulus-responsive IKK- and βTRCP-dependent ubiquitin-proteasome system (UPS) pathway, and a constitutive degradation pathway pertaining to free IκB, not bound to NFκB (ref. 28). In the case of IκBα, this pathway is solely dependent on the 26S proteasome^29^. However, for IκBɛ, the constitutive degradation pathway has not been characterized to the best of our knowledge.

In this study, we have investigated the roles of REGγ in inflammation response, DSS-induced colitis and CAC development using mouse models. REGγ promotes colitis and CAC, which are associated with increased NFκB activity. We identify IκBɛ as a functionally relevant target of REGγ-dependent, ubiquitin-independent degradation in colon epithelia, as evidenced by suppression of the *REGγ* knockout (KO) phenotype in doubly deficient mice.

## Results

### *REG γ* deficiency alleviates DSS-induced colitis

**Figure 1:**
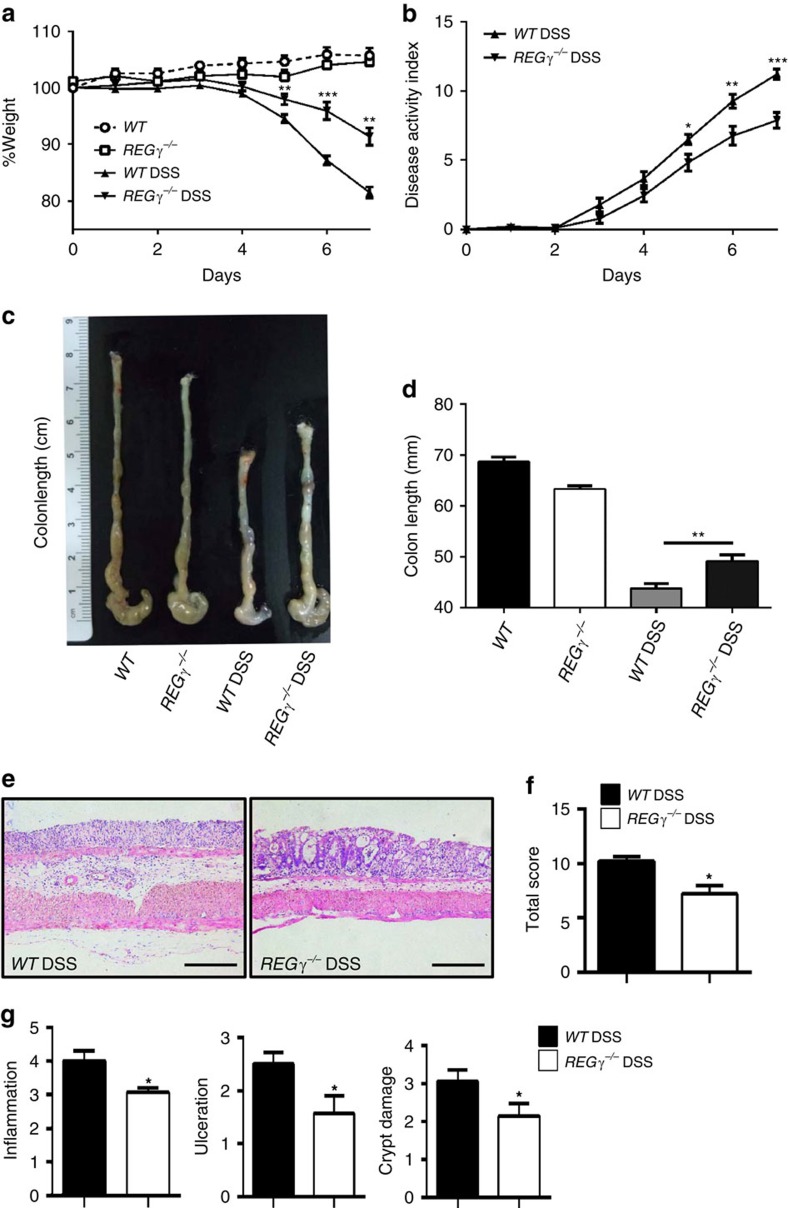
*REGγ*-deficiency attenuates the development of DSS-induced colitis. (**a**,**b**) Body weight (**a**) and DAI (**b**; weight loss, stool consistency and rectal bleeding scores) were recorded daily. *n*=6, normal group; *n*=9, DSS group. One representative experiment from three repeats is depicted. Data represent means±s.e.m. *P<0.05; **P<0.01; ***P<0.001, Student's *t*-test. (**c**,**d**) Mice were killed on day 7 after DSS treatment and colon lengths were quantitated depicting a representative experiment from three repeats. *n*=3,control group,; *n*=11, DSS group. **P<0.01, Student's *t*-test. (**e**) Histopathology of distal colon tissues collected at day 7 was examined by H&E staining. Representative images were shown. Scale bars, 200 μM. (**f**) Composite score of histopathology (inflammation, crypt damage plus ulceration scores). *n*=6 per group. Data represent means±s.e.m. of a representative experiment with three repeats.*P<0.05, Student's *t*-test. (**g**) Colon crypt damage, ulceration and inflammation were each individually scored for the mice in (**f**). *n*=6 per group. Data represent means±s.e.m. *P<0.05, Student's *t*-test.

To define the role of REGγ in intestinal inflammation, male *REGγ*^−/−^ mice were supplied with 2% DSS in drinking water for 7 days and monitored by body weight, stool consistency, rectal bleeding and colon length at day 7 (refs 15, 30). The disease progression and clinical scores in wild-type (WT) and *REGγ*-deficient mice were dramatically different. WT mice suffered from significant loss of body weight (Fig. 1a), diarrhoea and rectal bleeding (Fig. 1b), with reduced colon length (Fig. 1c,d). Histological analysis of colitis tissues from day 7 diseased mice by haematoxylin and eosin (H&E) staining revealed that *REGγ*^−/−^ mice had less crypt damage, ulceration and inflammation than *WT* littermates (Fig. 1e), as described in semi-quantitative scoring of histopathology (Fig. 1f,g). These results demonstrate that *REGγ*-deficiency can increase resistance to experimental colitis.

**Figure 2:**
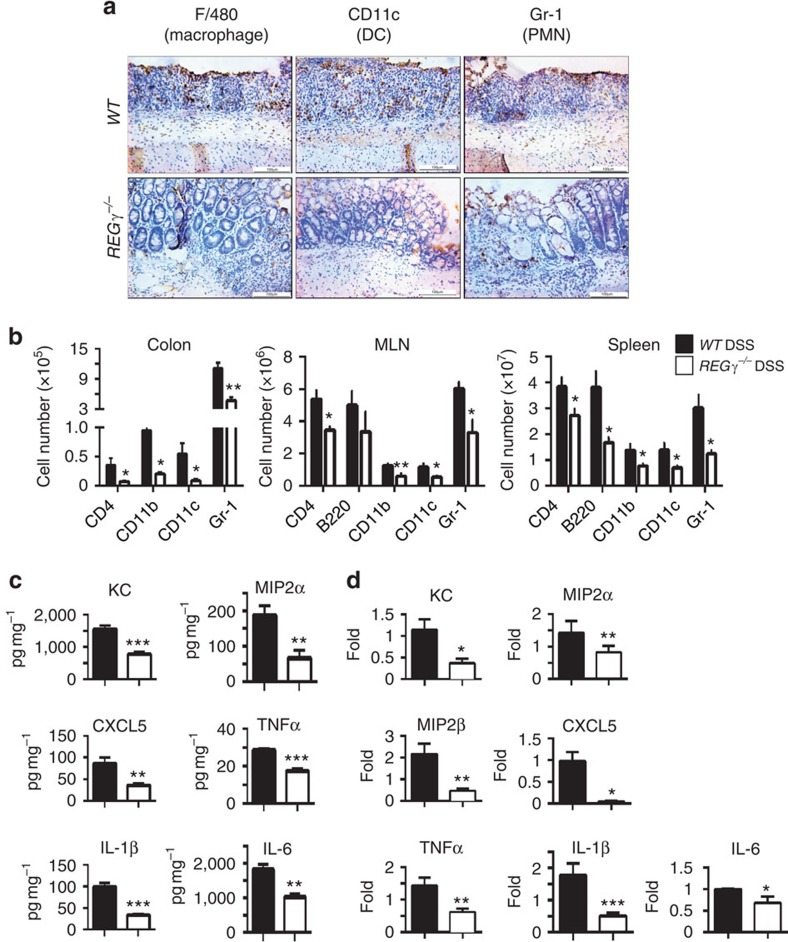
Reduced colon inflammation and production of cytokines and chemokines in DSS- treated *REGγ*^−/−^ mice. (**a**) Colon tissues from 7 days post-DSS mice were evaluated for infiltration of dendritic cells, macrophages and PMN by immunohistochemical staining with specific markers. Images are from one representative experiment of three repeats. Scale bars, 100 μM. (**b**) Colonic lamina propria mononuclear cells, MLN cells and splenocytes were analysed by flow cytometry after staining for CD4, CD11b, CD11c and Gr-1. Total numbers of CD4^+^, CD11b^+^, CD11c^+^ and Gr-1^+^ cells from day 7 lesions were calculated. *n*=6 per group. Data represent means±s.e.m. *P<0.05; **P<0.01, Student's *t*-test. (**c**) Colonic tissue explants were harvested at experimental day 7, cultured *ex vivo* for 24 h. Secreted cytokines were assessed from supernatants by BioPlex Multiplex and ELISA. *n*=10 per group. Data represent means±s.e.m. from three independent experiments. *P<0.05; **P<0.01; ***P<0.001, Student's *t*-test. (**d**) Colon epithelial cell were isolated at day 7, total RNA was extracted for expression analysis of related chemokines and cytokines by real-time RT–PCR. *n*=5 per group. Data represent means±s.e.m. *P<0.05; **P<0.01; ***P<0.001, Student's *t*-test.

To assess the severity of mucosal inflammation in the DSS-treated animals, we stained colon sections for polymorphonuclear neutrophils (PMN), macrophages and dendritic cells. WT colon lesions displayed much more infiltration of macrophages, PMNs and dendritic cells (Fig. 2a) than that in *REGγ*^−/−^ colitis tissues. To evaluate the scope of immune cell types affected in the colitis mouse models, we isolated myeloid cells in the colonic lamina propria from day 7 diseased mice and performed flow cytometry analysis. The numbers of all analysed myeloid cells (CD11b^+^, F4/80^+^, CD11c^+^ and Gr-1^+^) were significantly lower in *REGγ*^−/−^ mouse colons than those in WT colitis colons (Fig. 2b). Among these myeloid cells, neutrophils were the dominant cell type found in the lamina propria from *WT* and *REGγ*^−/−^ mice, with three times more cells in WT colons (Fig. 2b). More immune cells (with equal baseline values, Supplementary Fig. 1A) were seen to be infiltrated in WT spleens and mesenteric lymph nodes than in *REGγ*^−/−^ tissues, although B220^**+**^cells in mesenteric lymph node did not show significant changes (Fig. 2b). Together, these results indicate that REGγ plays a critical role in DSS-induced colitis.

### *REGγ* primarily affects colon epithelial cells in DSS-models

Consistent with alleviated colitis in *REGγ*^−/−^ mice, ELISA analysis of colonic explants revealed less production of pro-inflammatory cytokines and chemokines including KC, MIP2α, CXCL5, IL-1β, IL-6 and TNFα in *REGγ*^−/−^ mice than in WT counterparts (Fig. 2c). PCR with reverse transcription (RT–PCR) analysis showed that expression patterns of these pro-inflammatory cytokines and chemokines were similar to those observed by ELISA analysis (Fig. 2d) during acute colitis phase. In agreement with the *in vivo* data, similar gene expression profiles were observed in a human colon epithelial cell line HCT116 with or without *REGγ* stable knockdown (*shR* and *shN*) in the presence of TNFα (Supplementary Fig. 2A). Together, these results imply that the dampened inflammatory response in *REGγ*-deficient mice is closely related to changes in colon epithelial cells, leading to induction and progress of colitis.

**Figure 3:**
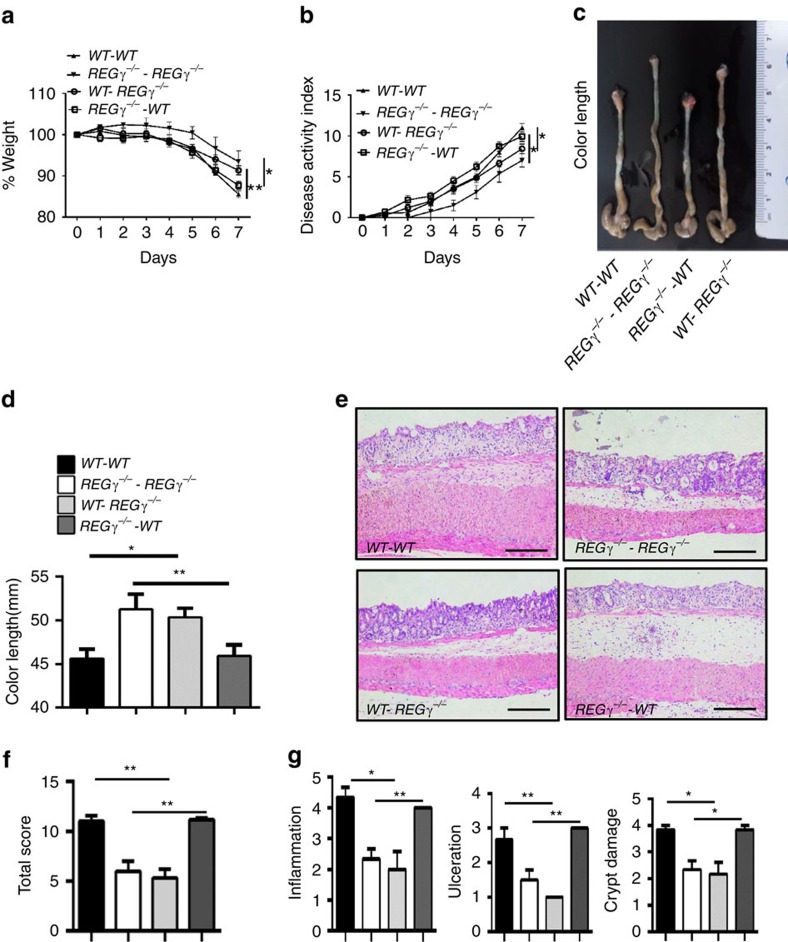
REGγ prominently regulates non-haematopoietic cells contributing to experimental colitis. (**a**) Body weight changes in bone marrow transplanted chimera mice. Data are derived from three independent experiments with *n*=6 (*WT*–*WT* group), *n*=6 (*WT*-*REGγ*^−/−^ group), *n*=7 (*REGγ*^−/−^-*WT* group) or *n*=7 (*REGγ*^−/−^-*REGγ*^−/−^ group) total, shown in means±s.e.m. *P<0.05; **P<0.01, Student's *t*-test. (**b**) Composited DAI scores. *n*=6/*WT*–*WT* group, *n*=6/*WT*-*REGγ*^−/−^ group, *n*=7/ *REGγ*^−/−^-*WT* group and *n*=7/*REGγ*^−/−^-*REGγ*^−/−^ group. Data represent means±s.e.m. from three independent experiments. *P<0.05, Student's *t*-test. (**c**) Mice were killed at experimental day 7 and a representative colon length in each group was displayed. (**d**) Statistic analysis of colon lengths. *n*=6/*WT*–*WT* group, *n*=6/*WT*-*REGγ*^−/−^ group, *n*=7/*REGγ*^−/−^- *WT* and *n*=7/REGγ^−/−^*REGγ*^−/−^-*REGγ*^−/−^ group. Data represent means±s.e.m. from three independent experiments. *P<0.05; **P< 0.01, Student's *t*-test. (**e**) Histopathological changes in distal colon tissues collected at day 7 by H&E staining. Representative images were shown. Scale bars, 200 μM. (**f**) Composited HAI. *n*=3 per group. Data represent means±s.e.m. of one representative experiment with three repeats. **P< 0.01, Student's *t*-test. (**g**) Individual histopathology. *n*=3 per group. Data represent means±s.e.m. for the mice in (**f**). *P<0.05; **P<0.01, Student's *t*-test.

To distinguish between the contributions of haematopoietic and non-haematopoietic cells to colitis progression in *REGγ* mouse models, we initiated bone marrow transplantation experiments. Bone marrow cells collected from *WT* or *REGγ*^−/−^ mice were transferred into lethally irradiated *WT* or *REGγ*^−/−^ recipient mice. After a 2 months reconstitution phase to achieve near-complete reconstitution of the haematopoietic system, recipient mice were administered with DSS for comparative analyses of colitis phenotypes. WT mice that received transfers from WT or *REGγ*^−/−^ bone marrow cells (*WT–WT* or *KO–WT*) showed similar susceptibility (Fig. 3a,b) and comparable colon length (Fig. 3c,d). WT recipients (*WT–WT* or *KO–WT*) demonstrated significantly higher disease scores than *REGγ*-deficient recipients (*WT–KO* or *KO–KO*). Histological and immunological analysis (Fig. 3e–g) also confirmed our observation that *WT* recipient mice (*WT*–*WT* or *KO*–*WT*) were more susceptible to DSS-induced colitis than *REGγ*^−/−^ mice (*WT*–*KO* or *KO*–*KO*). These results suggest that REGγ in non-haematopoietic cells (intestinal epithelial and stromal cells, for example, fibroblasts^31^,^32^ has a dominant contribution to DSS-induced colitis in the recipient hosts. Interestingly, *WT* mice or *REGγ*^−/−^ mice that received WT bone marrow grafts exhibited slightly more inflammation than those that received bone marrow from *REGγ*^−/−^ mice (*KO*–*WT* and *KO*–*KO*) (Fig. 3a–g), suggesting a partial contribution from the haematopoietic compartment. In summary, the roles of REGγ in DSS-induced colitis appeared to be mediated by both haematopoietic- and non-haematopoietic cells, but more prominently by the non-haematopoietic compartment.

### Reciprocal regulation of REGγ and NFκB in colon epithelium

**Figure 4:**
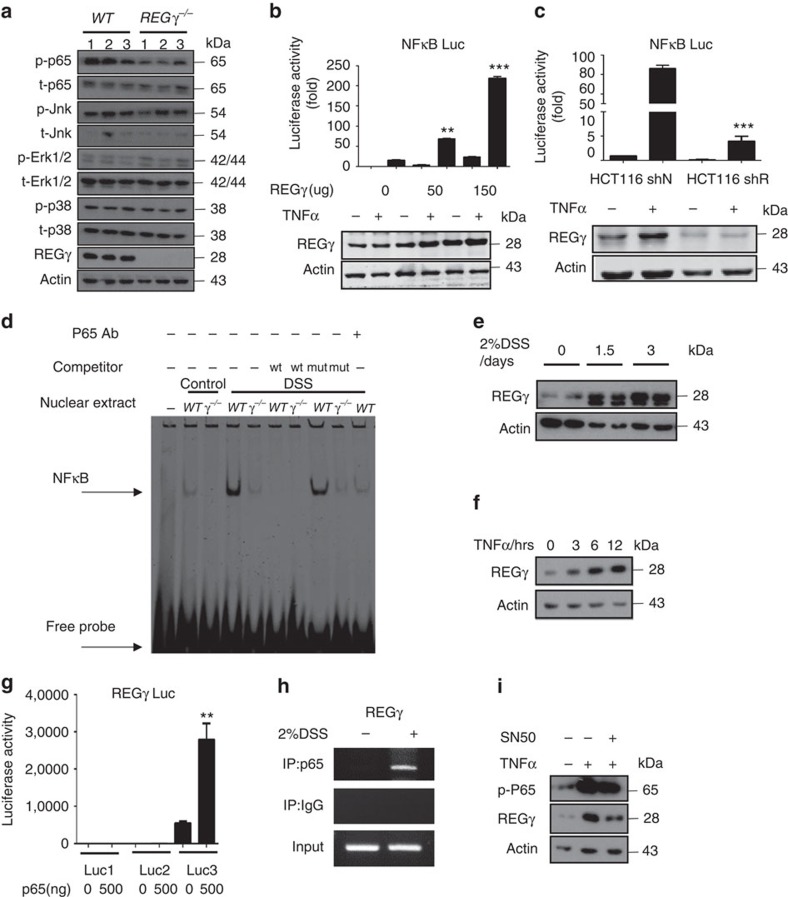
Reciprocal regulation of REGγ and NFκB pathway in colon epithelial cells. (**a**) Colon epithelial cells collected from *WT* or *REGγ*^−/−^ mice at 7 days after DSS administration were examined for activation of NFκB and MAPKs by western blot analysis. Each lane represents a sample from an individual mouse. (**b**,**c**) NFκB luciferase reporter activities were measured upon indicated REGγ overexpression (**b**) or knockdown (**c**) in HCT116 cells before and after 6 h TNF stimulation. *n*=3 per group. Data represent means±s.e.m. from three independent experiments. **P<0.01; ***P<0.001, Student's *t*-test. (**d**) Electrophoretic mobility shift assay using nuclear extracts of colon epithelial cells from *WT* or *REGγ*^−/−^ mice challenged with 2% DSS for 0 day or 7 days. Oligos containing NFκB consensus binding site were used as a probe. Representative image are from one representative experiment of the two repeats. (**e**,**f**) Expression of REGγ was detected in colon epithelial cells isolated from mice after 2% DSS administration for 0, 1.5 and 3 days (**e**) and in HCT 116 cells after indicated TNFα stimulation (**f**). Representative images were from one representative experiment of three repeats. (**g**) *REGγ-luciferase* reporter containing the cluster-3 NFκB binding elements was responsive to p65-mediated transcriptional regulation. *n*=3 per group. Data represent means±s.e.m. from three independent experiments. **P< 0.01, Student's *t*-test. (**h**) ChIP assays on *REGγ* promoter were performed in colon epithelial cell of mice after 2% DSS administration for 3 days. Representative of three repeats. (**i**) NFκB inhibitor, SN50, attenuated TNFα-mediated elevation of *REGγ*. Representative data were from three repeats.

To define the differentially expressed inflammation mediators in colon epithelia from WT and *REGγ*^−/−^ mice, we examined various signalling molecules that are closely related to inflammatory responses, including Erk, p38, JNK and NFκB in colon epithelial cells isolated from mice with DSS-induced colitis. We found significant reduction in p-p65, but not other molecules, in *REGγ*^−/−^ colon epithelial cells compared with those from *WT* (Fig. 4a), suggesting that REGγ may positively regulate the NFκB pathway. Similar results of p-p65 elevation were found in a human colon epithelial cell line compared with the *REGγ* knockdown controls (Supplementary Fig. 2B). To validate REGγ-dependent regulation of p65 signalling, NFκB luciferase reporter activity was measured upon *REGγ* overexpression or depletion in HCT116 cell before and after TNF stimulation. Expectedly, the increase of NFκB luciferase activity correlated proportionally with the rise of REGγ levels (Fig. 4b), whereas the NFκB reporter activity decreased when *REGγ* was knocked down (Fig. 4c). These findings were consistent with previous studies that NFκB signalling in colon promotes inflammation. Moreover, electrophoretic mobility shift assay exhibited reduced DNA-binding ability of NFκB in nuclear extracts from *REGγ*^−/−^ colon epithelial cells compared with that from the WT (Fig. 4d). Chromatin immunoprecipitation (ChIP) assay found reduced recruitment of NFκB/p65 to the IL8 gene promoter in the stable *REGγ*-knockdown cells (Supplementary Fig. 2C). Together these data indicate that REGγ can positively regulate the NFκB pathway in colon epithelial cells.

Interestingly, REGγ levels in WT epithelial cells were progressively increased upon DSS stimulation (Fig. 4e). To test whether NFκB may regulate *REGγ* in a positive-feedback fashion, we treated HCT116 cells with TNFα for various amounts of time. Strikingly, expression of REGγ was increased with the duration exposure to TNFα (Fig. 4f). We verified that TNFα treatment also enhanced the expression of *REGγ* transcripts in colon explants (Supplementary Fig. 2D). To understand whether NFκB may directly regulate *REGγ* transcription, we searched putative NFκB-binding elements throughout ∼2 kb sequences upstream of *REGγ* promoter. Three clusters of NFκB-binding elements identified via bioinformatic analysis were cloned into the basal *REGγ*-*Luc* reporter (Supplementary Fig. 2E). Only the *REGγ*-*Luc3* reporter containing NFκB-binding elements within −454 to −252 had elevated activity upon co-expression of p65 (Fig. 4g), suggesting that *REGγ* gene is a transcription target of the NFκB pathway. Indeed, genome-wide p65 ChIP-seq analysis in MEFs stimulated with TNF/LPS revealed a strong peak in the promoter-proximal region of the *REGγ* gene (Supplementary Fig. 2F). To validate *in vivo* binding of NFκB to the *REGγ* promoter in colon epithelial cells, we performed ChIP assays using primers flanking the cluster-3 NFκB-binding elements. Colon epithelial cells isolated from 3-day DSS-treated mice showed significant recruitment of p65 to the *REGγ* promoter (Fig. 4h), substantiating NFκB-dependent regulation of the *REGγ* gene. Furthermore, application of an NFκB inhibitor, SN50, attenuated TNFα-mediated elevation of REGγ (Fig. 4i, p-P65 served as a control and Supplementary Fig. 2D), suggesting that a reciprocal regulation loop exists between NFκB and the proteasome-activator REGγ.

### REGγ regulates NFκB activity by degrading IκBɛ

To elucidate the molecular mechanism by which REGγ regulates the NFκB pathway, we carried out a high-throughput proteomic screen of potential REGγ targets using antibody arrays (FullMoon BioSystems). Among the proteins differentially expressed in *REGγ*^*+/+*^ and *REGγ*^−/−^ MEF cells, the positive controls, known REGγ targets, p53 and p21 (refs 17, 33), were expectedly higher in *REGγ*^−/−^ MEFs (Supplementary Table 1). In agreement with our findings, p-p65 levels in these antibody arrays were significantly higher in *REGγ*^*+/+*^versus *REGγ*^−/−^ MEF cells (Supplementary Table 1). Furthermore, a member of IκB family, IκBɛ, was markedly diminished in *REGγ*^*+/+*^ compared with *REGγ*^−/−^ MEFs (Supplementary Fig. 3A and Supplementary Table 1), although it is a known NFκB target gene. *IκBɛ* is known to regulate post-induction attenuation of RelA and cRel-containing NFκB dimers, but to date no non-haematopoietic physiological function has been ascribed to it. Our results are consistent with a role for *IκBɛ* in dampening NFκB activity in colon epithelial cells and attenuating the progress in colitis; our data suggest that REGγ neutralizes inhibition of inflammation by triggering IκBɛ degradation.

**Figure 5:**
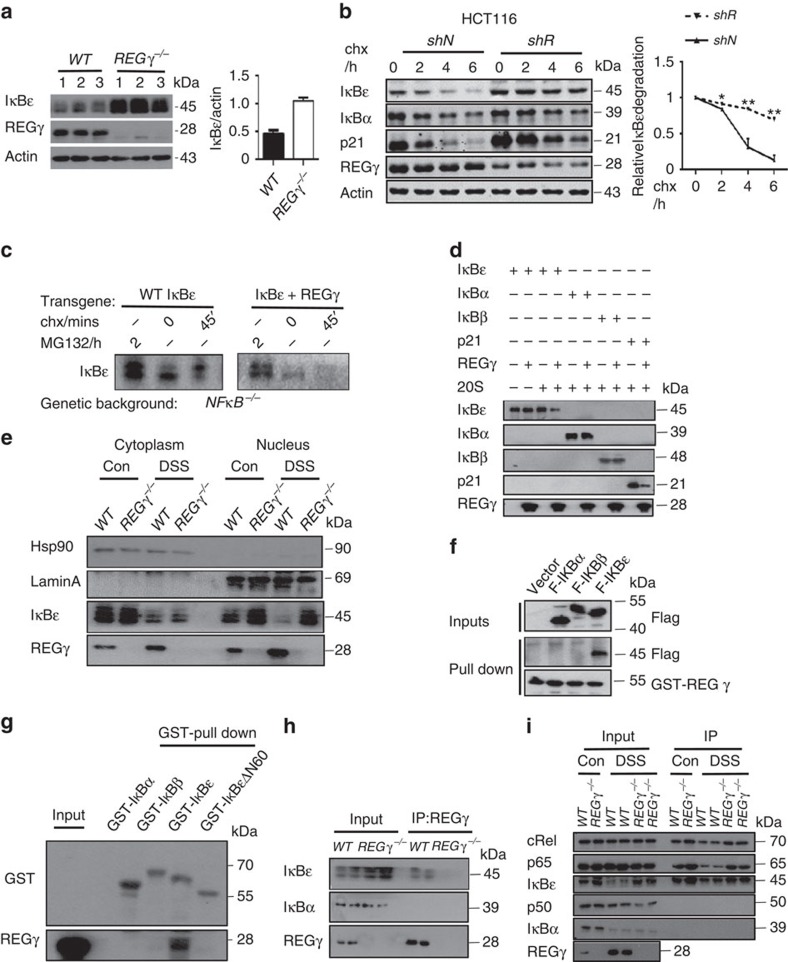
REGγ interacts with IκBɛ and promotes its degradation. (**a**) Expression of IκBɛ in colon epithelial cells isolated from *WT* and *REGγ*^−/−^ mice following 7 days of DSS treatment. Representative of four repeats (left). Densitometric analysis of IκBɛ relative to actin protein. Data represent means±s.e.m.; *n*=10 per group; ***P<0.001 (right). (**b**) HCT116 *REGγ* shR or *shN* control cells were treated with cycloheximide (100 μg ml^−1^) for indicated times followed by western blotting. Representative images are from three repeats (left). Densitometric analysis of relative IκBɛ degradation. Data represent means±s.e.m.; *P<0.05, **P<0.01(right). (**c**) Immunoblot for HA-tagged IκBɛ with or without REGγ expressed from retroviral transgenes in NFκB-deficient cells. Representative images are from three repeats. (**d**) REG γ mediates proteolytic degradation of IκBɛ in a cell-free system. Purified REG γ, 20S proteasome, and *in vitro*-translated IκBɛ, IκBα and IκBβ were incubated as indicated and described in Methods followed by western blot analysis. Representative images are from three repeats. (**e**) IκBɛ accumulates in nucleus in REG γ-deficient colon epithelial cells. Colon epithelial cells collected from *WT* or *REGγ*^−/−^ mice at 0, 7 days post DSS administration were examined for IκBɛ after cytoplasm and nucleus separation by western blot analysis. Representative results were from two repeats. (**f**) Interactions between REGγ and IκBs. Pulldown assays were performed with 293T cells lysis transiently transfected flag- IκBɛ, IκBα or IκBβ and GST-REGγ. Representative of three repeats. (**g**) Interaction specificity between REGγ and IκBɛ. Purified REGγ and IκBs or IκBɛΔN60 were used in GST-pulldown analysis as described in Methods. Representative of four repeats. (**h**) Interactions between REGγ and IκBɛ in murine colon epithelial cell after 3 days DSS administration. Proteins were IP with an anti-REGγ Ab. Representative images were from three repeats. (**i**) Colon epithelial cells collected from *WT* and *REGγ*^−/−^ mice with or without prior exposure to DSS were IP with IκBɛ antibody and immunoblotted as indicated. Representative of two repeats.

To validate the changes of *IκBɛ* observed in antibody array analysis, we measured its protein levels in colon epithelial cells isolated from *WT* and *REGγ*^−/−^ mice following 7 days of DSS administration. We found a significantly higher expression of IκBɛ, but not IκBα or IκBβ, in *REGγ*^−/−^ colon epithelial cells (Fig. 5a and Supplementary Fig. 3B). Consistently, stable knockdown of *REGγ* in HCT116 (*shR*), resulted in an elevation of IκBɛ (Fig. 5b), indicating a strong negative correlation between REGγ and IκBɛ protein levels. In the presence of cycloheximide to inhibit *de novo* protein synthesis, IκBɛ decayed much faster in HCT116 control cells (*shN*) than in *shR* cells (Fig. 5b), although *REGγ*-deficiency had a negative effect on IκBɛ transcript levels (Supplementary Fig. 3C). Given that the degradation of NFκB-bound IκBs has been characterized as mediated by the E3 ligase βTRCP and the UPS, we analysed how IκBɛ might be regulated by REGγ. We utilized cells deficient in the three canonical NFκB components. In contrast to IκBα, and previous suggestions (O'Dea and Hoffmann^25^), we found that IκBɛ is in fact long-lived in fibroblasts. However, upon overexpression of REGγ the half-life of IκBɛ is dramatically shortened (Fig. 5c), indicating that IκBɛ is subject to degradation by the REGγ-degradation pathway. To determine whether the effect of REGγ on IκBɛ degradation is direct or indirect, we examined the activity of REGγ in cell-free proteolysis. Incubation of *in vitro* translated IκBs with 20S proteasome or purified REGγ alone showed no significant degradation of *IκBɛ*. However, a combination of REGγ and 20S proteasome promoted marked degradation of *IκBɛ* in the absence of additional ATP, with no significant effect on IκBα or IκBβ (Fig. 5d).

To address *REGγ*-dependent degradation of IκBɛ in more detail, we performed cell fractionation experiments to understand where the REGγ-proteasome primarily degrades IκBɛ. Our data showed some accumulation of IκBɛ in the nuclear fractions from *REGγ*^−/−^ colon epithelial cells (Fig. 5e), suggesting that *IκBɛ* maybe mainly degraded in the compartment where *REGγ* is mostly localized. Less difference of *IκBɛ* in cytosol was probably due to activation of NFκB and ubiquitin-dependent degradation of IκBɛ.

Moreover, we analysed molecular interactions between REGγ and IκBs by co-immunoprecipitation. We found that only IκBɛ, but not IκBα or IκBβ, could be immunoprecipitated (IP) by GST-REGγ (Fig. 5f). To determine the recognition specificity by REGγ, we analysed amino acid sequences among the three IκBs (Supplementary Table 2). It appears that the major differences among these IκBs are within the N-terminal 60 amino acids. We generated an IκBɛ construct with deletion of the N-terminal 60 amino acids (GST-IκBɛΔN60). GST pulldown experiments using the GST-IκBɛΔN60 suggested the binding of REGγ to IκBɛ via its N-terminus (Fig. 5g). As expected, *in vivo* interactions between REGγ and IκBɛ were observed in DSS-treated colon epithelial cells (Fig. 5h). Taken together, we conclude that REGγ directly interacts with IκBɛ and promotes non-ATP and non-ubiquitin-dependent degradation of IκBɛ.

As a negative regulator for RelA and cRel-containing NFκB dimers^34^, IκBɛ associates with different Rel proteins in a cell-specific manner^35^. To understand how REGγ regulates the composition of the NFκB complexes associated with IκBɛ, we collected colon epithelial cells from WT and *REGγ*^−/−^ mice or HCT116 *shN* and *shR* cells for immunoprecipitation analysis using an anti-IκBɛ antibody. The results indicate that IκBɛ form a complex with cRel and p65, but not p50 and *REGγ* deficiency significantly stabilizes the IκBɛ complexes (Fig. 5i and Supplementary Fig. 3D). Interestingly, alteration in REGγ levels has profound impact on cytoplasmic and nuclear IκBɛ equilibrium (Fig. 5e), which contributes to modification of NFκB activity. To recapitulate this by visualizing the impact of REGγ/IκBɛ on p65 cellular localization, we transfected GFP-IκBɛ into HCT116 *shN* or *shR* (*REGγ* deficient) cells and scored for p65 positive nuclear/cytoplasmic ratios. While untransfected *shN* cells had a dramatically higher nuclear/cytoplasmic ratio for p65 with TNFα treatment, the *shN* cells transfected with exogenous IκBɛ had a similarly reduced nucleocytoplasmic ratio for p65 localization as in HCT116 *shR* cells (Supplementary Fig. 3E,F), indicating a regulation of p65 activity by the *REGγ-IκBɛ* pathway.

### *REGγ*/*IκBɛ* double-KO restores colitis severity

**Figure 6:**
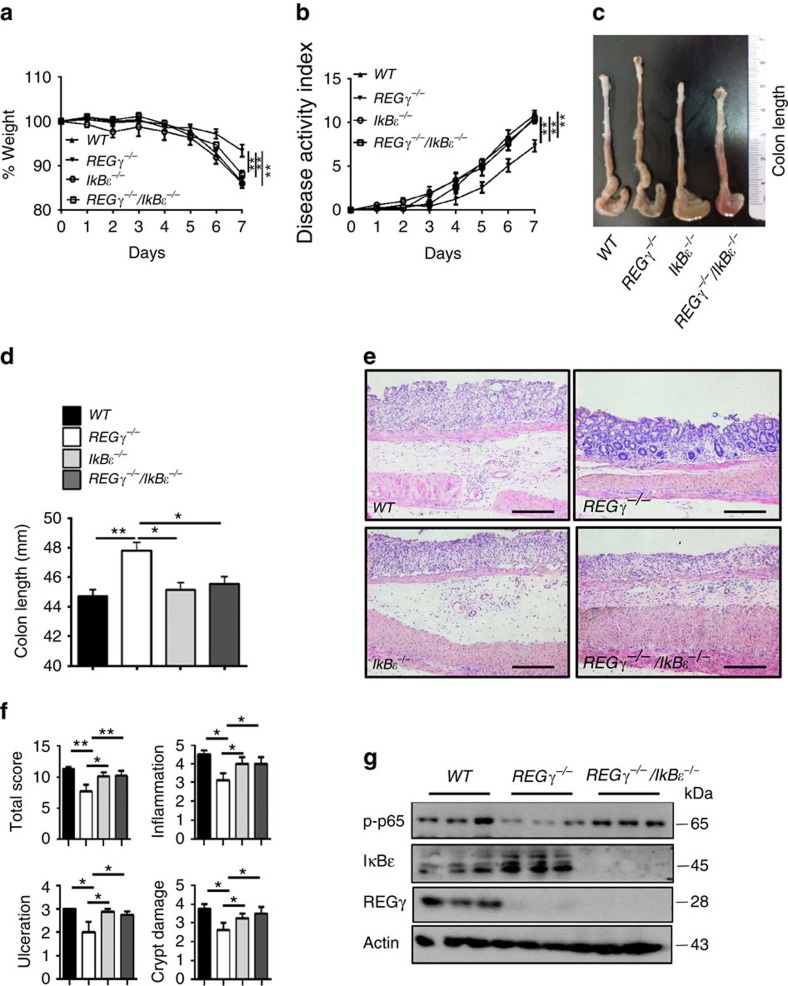
Mice with double deletion of *REGγ* and *IκBɛ* exhibit severe colitis phenotypes. (**a**,**b**) Body weight (**a**) and Composited DAI scores (**b**) were recorded daily. *n*=10, WT and double-KO group; *n*=9, *REGγ*^−/−^ group; and *n*=7, IκBɛ^−/−^ group. Data represent means±s.e.m. from three independent experiments. *P<0.05, Student's *t*-test. (**c**,**d**) Mice were killed on day 7 after DSS administration. Representative colon length was shown in **c** and quantitated results for colon length in each group of mice were displayed (**d**). *n*=6 per group. Data represent means±s.e.m. from three independent experiments. *P<0.05; **P<0.01, Student's *t*-test. (**e**) Histopathological changes were examined by H&E staining. Representative images of distal colon section were from three repeats. Scale bars, 200 μM. (**f**) Colon crypt damage, ulceration and inflammation were scored individually, and composite HAI was scored. *n*=3 per group. Data represent means±s.e.m. of one representative experiment from three repeats. *P<0.05, Student's *t*-test. (**g**) Restoration of NFκB activity in double-KO colon epithelial cells. Each lane represents a sample from an individual mouse.

In view of our findings that DSS-induced colitis is relieved in *REGγ*-deficient mice, with augmented IκBɛ accompanied by reduced NFκB activity in colon epithelial cells, we wondered whether REGγ aggravates DSS-induced colitis mainly through degrading IκBɛ, which otherwise, in *REGγ*-deficient mice functions to inhibit colitis progression. To test this hypothesis, we generated *REGγ/IκBɛ* double-KO mice by crossing *RE*Gγ^−/−^ with *IκBɛ*^−/−^ mice, and then induced colitis in *WT*, *REGγ*^−/−^, *IκBɛ*^−/−^ and the double-KO mice. Interestingly, the compound deficiency nearly abolished the protection against colitis observed in *REGγ*^−/−^ mice. The *REGγ/IκBɛ* double-KO and WT mice had comparable indices of colitis, particularly in terms of loss of body weight (Fig. 6a) and disease activity index (DAI) (Fig. 6b). The above findings also were reflected by the gross appearance of the colon. The colon lengths in WT and *REGγ/IκBɛ* double-KO mice were significantly shorter than in *REGγ*^−/−^ mice (Fig. 6c,d). Histologically, colon sections had more striking ulceration, inflammation and crypt damage in both WT and *REGγ*/*IκBɛ* double-KO than in *REGγ*^−/−^ mice (Fig. 6e,f). Moreover, colon tissues collected from the double-KO mice produced much more pro-inflammatory chemokines than those from *REGγ*^−/−^ mice (Supplementary Fig. 4A). Compared with *REGγ*^−/−^, colon epithelial cells from WT and *REGγ*/*IκBɛ* double-KO mice had dramatically higher expression of p-p65 (Fig. 6g), consistent with the p-p65 results observed in HCT116 *shN*, *shR* and *dKD* (*REGγ* and *IκBɛ* stable knockdown) cells (Supplementary Fig. 4B), supporting the contribution of NFκB activity to colitis. In summary, protective role of REGγ inhibition in DDS-induced colitis is dependent on IκBɛ.

### REGγ is elevated in both mouse and human colitis

**Figure 7:**
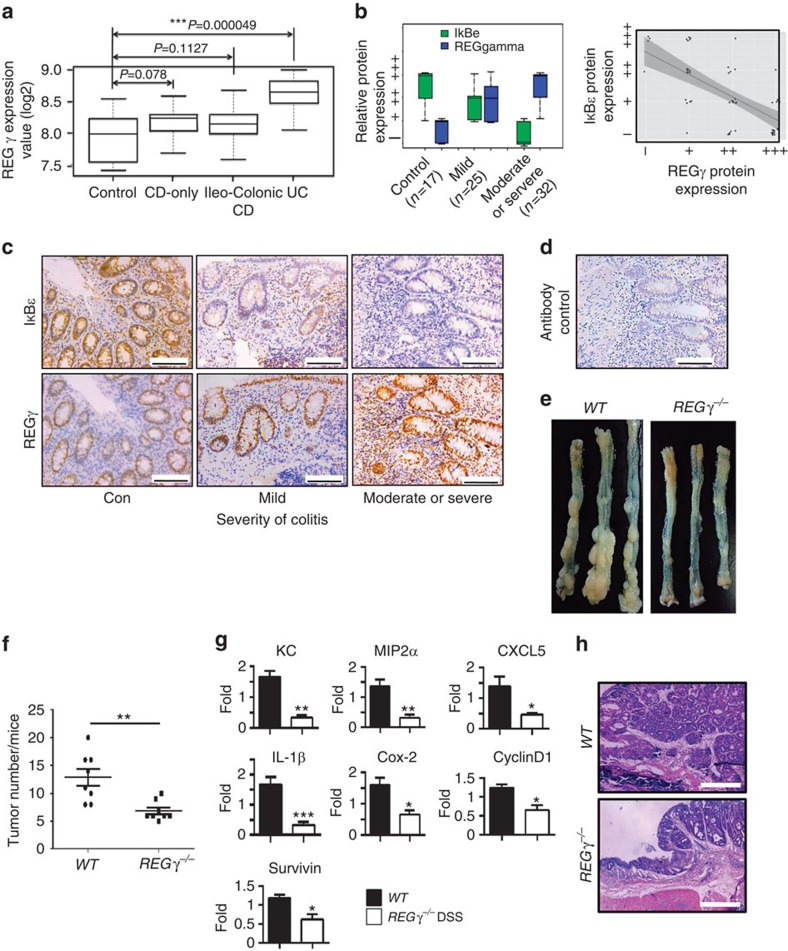
*REGγ* overexpression correlates with severity of colitis and promotes tumourigenesis in colon. (**a**) Boxplot of *REGγ* expression values (log2) among four groups of microarray data (healthy control, CD-only, Ileo-Colonic CD and UC). Median values are indicated by the transverse line within the box. (**b**) Correlation between REGγ or IκBɛ protein expression and severity of colitis in controls and patients with UC (left). REGγ expression levels (−∼+++) and IκBɛ Expression levels (−∼+++) were evaluated as described in Methods and were visualized by ggplot2 packages with R language. Correlation between REGγ and *IκBɛ* protein expressions in controls and patients with UC is displayed (right). By scatter plots and boxplots analysis, correlation between REGγ and IκBɛ were analysed across all the groups. The Pearson correlation is −0.69, *P* values<0.001(***). All statistical analysis was performed in R.The number of patient samples and controls were indicated. (**c**) Representative IHC analysis of REGγ and IκBɛ expression in control and UC patients. Scale bars, 100 μm. (**d**) An IgG control for IHC is shown. (**e**) Appearances of representative CAC in *WT* and *REGγ*^−/−^ mice. (**f**) The number of tumours in the colons from *WT* and *REGγ*^−/−^ mice was quantitated. One representative experiment (*n*=8 each group) of three repeats is depicted. **P<0.01,Student's *t*-test. (**g**) Quantitative RT–PCR analysis of CXCL1, MIP2α, MIP2β, CXCL5, IL-1β, COX-2, Survivin and Cyclin D1 expression in the colon tumours. *n*=6 per group. Data represent means±s.e.m. from one representative result of three repeats. *P<0.05; **P<0.01; ***P<0.001, Student's *t*-test. (**h**) Representative histology of tumours is shown. Scale bars, 500 μm.

Our previous finding that *REGγ* is overexpressed in colon cancer compared with normal tissues or adjacent non-cancer tissues^36^ prompted us to examine the expression of REGγ in experimental colitis tissues; a significantly higher level of REGγ was detected in colitis lesions than in normal controls (Supplementary Fig. 5A,B). Expression of *REGγ* mRNA was also increased after DSS induction (Supplementary Fig. 5C). We next addressed the correlation between *REGγ* expression and human IBDs. We carried out bioinformatics analysis of previously collected microarray data sets (ID:GSE10616) by statistical approaches as described in methods. The results indicate that *REGγ* RNA levels are significantly higher in UC specimens compared with healthy and CD controls (Fig. 7a). Moreover, we evaluated the correlation between REGγ expression and UC status by immunohistochemistry (IHC) analysis of 74 human colon tissues/lesions. The expression of REGγ in three groups of specimens was scored double-blindly and statistically analysed as described^36^. We detected significantly higher expression of REGγ in severe colitis cases (Fig. 7b,c) with an IgG control (Fig. 7d), suggesting a positive correlation between *REGγ* and UC inflammatory lesions. However, expression of IkBɛ in these severe cases had significant reduction (Fig. 7b,c), reflecting a negative relation between REGγ and IκBɛ in UC. Thus, we demonstrate that higher expression of REGγ is correlated with colitis, implicating an important role of REGγ in the development of colitis, for which REGγ may potentially serve as a maker.

### *REGγ* deficiency attenuates colitis-associated colon cancer

Since a link between inflammation and cancer has long been observed^9^,^10^, the protection of *REGγ*^−/−^ mice against colitis development suggests that REGγ also may promote colitis-associated tumourigenesis. To test the association of REGγ with colon tumour formation, we injected a single dose of the DNA-methylating agent AOM followed by a cycle of 2% DSS-administration (Supplementary Fig. 5D); intriguingly, *REGγ*^−/−^ mice had a lower tumour burden than WT mice (Fig. 7e). *WT* mice had 13 tumours per animal on average, whereas *REGγ*^−/−^ mice had seven (Fig. 7f). Importantly, *REGγ*^−/−^ mice had smaller tumours, with a majority of *REGγ*^−/−^ tumours being <2 mm in diameter compared with the >2 mm tumours in WT mice (Supplementary Fig. 5E). Expression of pro-inflammatory cytokines and chemokines, including KC, MIP2α, CXCL5 and IL-1β, was significantly lower in the colon of *REGγ*^−/−^ mice than in *WT* mice injected with AOM (Fig. 7g), which was consistent with histological analysis (Fig. 7h). Furthermore, AOM and DSS-treated *REGγ*^−/−^ mice had reduced expression of additional NFκB target genes, such as COX-2, cyclinD1 and survivin (Fig. 7g). In summary, our findings indicate that *REGγ* deficiency results in a less inflammatory micro-environment, attenuated intestinal epithelial cell proliferation and a reduced progression of tumourigenesis.

Given the link between elevated NFκB activity and inflammation-related tumourigenesis^37^,^38^,^39^, we next tested the role of IκBɛ in inflammation-driven colon tumourigenesis. Following tumour induction in mice with different genotypes, we found that both WT mice and *REG γ/IκBɛ* double-KO mice had more colon tumours than *REGγ*^−/−^ mice (Supplementary Fig. 5F,G). Moreover, the *REGγ/IκBɛ* double-KO mice had comparative tumour sizes with that observed in WT mice, both having larger tumours than *REGγ*^−/−^ mice (Supplementary Fig. 5H). Our results suggest that IκBɛ contributes to the protective role against colitis-associated tumourigenesis in *REGγ*^−/−^ mice by suppressing NFκB activity.

To validate the specific regulation of IκBɛ by REGγ, we performed a ‘rescue' experiment in *REGγ*/*IκBɛ* double-KOt mice. Simultaneous deletion of *REGγ* and *IκBɛ* nearly abolished the protection in *REGγ*^−/−^ mice for the development of colitis, suggesting a key role of *IκBɛ* in mediating REGγ function in our experimental system. Consistent with previous report that *IκBɛ*^−/−^ mice have no effect on NFkB activity^40^, we see little phenotypic changes in DSS colitis between WT and *IκBɛ*^−/−^. In fact, we found compensational increase of IkBα/IkBβ in *IkBɛ*^−/−^ cells and no significant changes in p-p65 compared with WT controls (Supplementary Fig. 6A), reminiscent of minimal constitutive NFκB in *IkB-α* KO MEFs (ref. 41). We reasoned that in response to DSS treatment, the REGγ-IkBɛ regulation may impinge upon an ‘acute take away' of IkBɛ, therefore, lead to an enhanced NFκB signalling. To validate this, we silenced *IκBɛ* with transient siRNA treatment (acute take away). This ‘acute depletion of *IκBɛ*' did not cause compensation increase of other IκBs, but induced significant elevation in p-p65 (Supplementary Fig. 6B).

## Discussion

**Figure 8:**
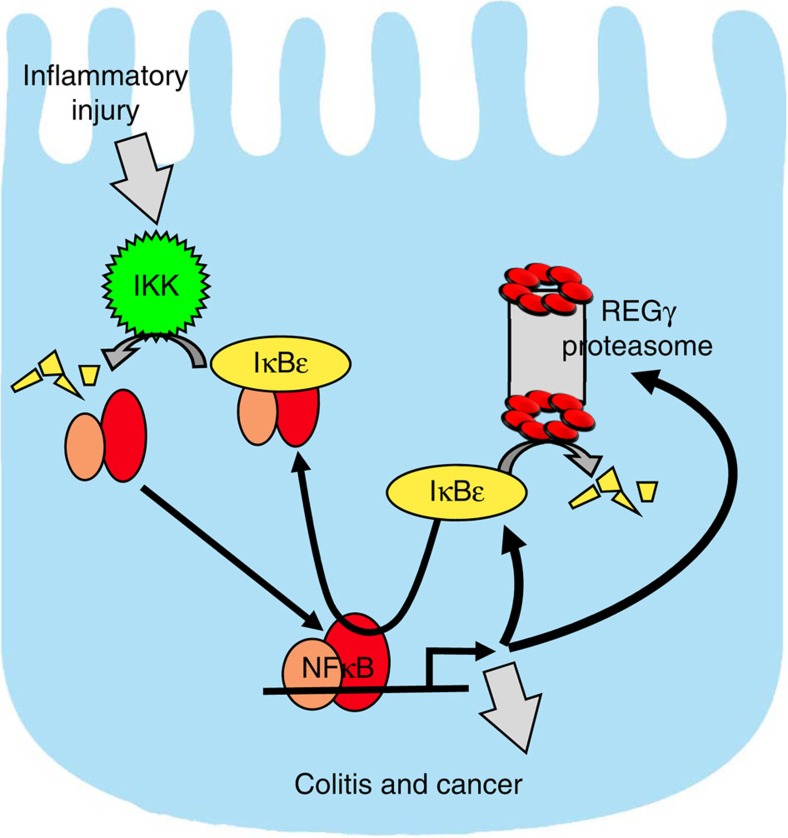
A schematic model depicts the reciprocal regulation between REGγ and NFκB. Inflammatory injury impairs integrity of the epithelial barrier, triggering initial activation of NFκB, which promotes expression of *IκBɛ* and *REGγ*. Elevated REGγ enhances degradation of IκBɛ protein in a ubiquitin-independent manner, neutralizing its ability to inhibit NFκB, thus leading to further elevation of REGγ and activation of NFκB. The reciprocal regulation between REGγ and NFκB via IκBɛ constitutes a novel regulatory circuit with the potential for positive feedback that can lead to run-away inflammation, the development of colitis and CAC. Notably, IκBɛ KO mice are not resistant to DSS colitis, unlike REGγ KOs. We speculate this is because of compensatory upregulation of other IκBs that takes place in the case of chronic IκBɛ deficiency (IκBɛ KO) but not acute deficiency such as in *REGγ*-deficient mice.

In this study, we demonstrate that the proteasome activator REGγ is a regulatory factor involved in bowel inflammation and CAC development in DSS models. Impaired integrity of the epithelial barrier and initial activation of NFκB-enhanced intestinal epithelial expression of *REGγ*, further enhance NFκB signalling by negatively regulating IκBɛ and promoting cytokine and chemokine expression (Fig. 8). Depletion of *REGγ* alleviates the severity of experimental colitis and CAC in an IκBɛ-dependent manner.

Deregulated cytokine production and signal transduction in intestinal epithelial cells (IECs), lymphocytes and macrophages have been implicated in the pathogenesis of IBD. The NFκB pathway has been appreciated as a key pathway in IBD development. Increased expression and activity of NFκB is well documented in the inflamed intestinal mucosa and macrophages of IBD patients^42^,^43^. Application of NFκB inhibitors, including curcumin and parthenolide, successfully attenuated bowel inflammation in animal models^44^,^45^, further demonstrating the central role of NFκB activation in the development of IBD (ref. 46). However, the mechanisms and roles of NFκB signalling in intestinal immunity remain less clear. Several studies suggest that NFκB activation may have an essential protective role against intestinal inflammation. For example, mice lacking NEMO in IEC (NEMO^IEC-KO^) displayed a spontaneous development of colon inflammation^47^. Furthermore, mice with IEC-specific ablation of IKK2 developed more severe DSS-induced colitis^38^ and had excessive production of pro-inflammatory Th1 cytokine with parasite infection^48^. These observations suggest that NFκB activation in epithelial cells is necessary for preserving intestinal immune homoeostasis, while other reports indicate constitutive NFκB activation may induce inflammation and tissue damage^49^,^50^. An important question is how to reconcile these seemingly contradictory observations. Perhaps maintaining NFκB activity within a normal range is crucial for natural defense against intestinal inflammation. Either too much or too little NFκB activation might be detrimental to the intestinal homoeostasis. Consistent with these notions, reciprocal positive-feedback regulation between REGγ and NFκB in IEC harbours the risk for run-away NFκB overactivation and development of bowel inflammation and CAC in DSS models. By analysing the correlation between *REGγ* and IBD in published database (ID:GSE10616), we found no significant changes in *REGγ* expression between CD and normal controls. However, the UC group had elevated *REGγ* expression, suggesting that CD and UC are two different forms of IBD.

The UPS plays an important role in the activation of the NFκB pathway. Ubiquitin-dependent degradation of IκBs is central to activating NFκB. However, we now further reveal that IκBɛ is subject to REGγ-proteasome-dependent degradation, which thereby neutralizes these protective effects in *WT* mice. So far, most of the REGγ target proteins can also be degraded by ubiquitin-dependent pathway. We have proposed that under physiological condition, REGγ mainly degrades target proteins to maintain relatively lower steady-state levels. Pathological increase of REGγ upon DSS treatment may tip the balance between ubiquitin-dependent and ubiquitin-independent degradation pathway.

Interestingly, no previous physiological functions in inflammation or innate immune defenses have been established for IκBɛ. Here we have defined KC, MIP2α and CXCL5 as IκBɛ-regulated genes in colon epithelial cells and elucidated REGγ as a specific regulator for IκBɛ (Supplementary Fig. 4C), expanding our knowledge to the IκBɛ regulatory pathway. Although *in vitro* immune responses of bone marrow-derived immune cells from WT and *REGγ*^−/−^ mice are not significantly different (Supplementary Fig. 7A–D), we did observe contributions from immune cells to the development of DSS colitis with *in vivo* model (Fig. 2), reflecting that REGγ affects both haematopoietic and non-haematopoietic compartments in the DSS model.

Homoeostasis of NFκB signalling requires a balance between positive and negative feedback regulatory network. The NFκB target genes, including IκBs and A20, play critical roles in termination of the active canonical NF-κB pathway^51^. In our study, NFκB induces *REGγ* to produce a positive-feedback upregulation of NFκB via REGγ-dependent degradation of IκBɛ. Because of specific binding between REGγ and N-terminus of IκBɛ (Fig. 5f), but not for other negative regulators such as IκBα, IκBɛ seems to be the only factor involved in the NFκB/REGγ regulatory loop. Identification of this non-canonical degradation pathway for IκBɛ may explain why the degradation dynamics are different between IκBɛ and IκBα. Given that IκBɛ shows slower regulatory dynamics than IκBα, it is likely that regulation of IκBɛ may be particularly relevant to control of homoeostatic or chronic inflammation. Since both *IκBɛ* and *REGγ* are NFκB-response genes, the resulting feedback circuit may function to provide constitutive control but is subject to pathological derailment in inflammatory colitis. Consistent with this notion, we found increased expression of REGγ in colon epithelial cells in experimental colitis as well as in specimen from human UC, partially interpreting the tissue-specific and cell-specific effects of the REGγ-proteasome action.

Abundant publications suggest that increased inflammation is closely associated with elevated NFκB signalling and tumourigenesis. We have recapitulated this scenario by demonstrating that *REGγ* depletion attenuates both intestinal inflammation and colon tumourigenesis. Our findings highlight a crosstalk between the canonical NFκB pathway and a non-canonical proteasome pathway that underlies the molecular mechanism in the development of IBD and intestinal tumour formation. By no means have we excluded the possibility that other REGγ target proteins (IκBɛ-independent mechanisms) may also be involved in the complexity of the experimental bowel disorder and CRC. Elevated p53 may explain why there is no significant differences in apoptosis between *WT* and *REGγ*^−/−^ colitis (Supplementary Fig. 7E), despite the known fact that IEC apoptosis contributes to the development of UC.

Given the key roles of NFκB hyperactivation in IBD, proper control of NFκB activity remains an attractive approach for the treatment of IBD. A variety of established anti-inflammatory agents including glucocorticoids, methotrexate and anti-TNFα antibodies are known to inhibit the NFκB pathway, at least in part. However, none of these components is specific for the NFκB pathway, and it may thus show variable responses. Given that both hyperactivation and absence of NFκB signalling are detrimental to intestinal health, direct inhibitors of IKK or NFκB may be risky. Furthermore, current proteasome inhibitors produce severe side effects, thus preventing wide clinical application. Our discovery of a specific REGγ-dependent pathway to tune NFκB activity via IκBɛ may provide an opportunity to develop new molecules targeting the non-canonical (11S Cap) proteasome degradation pathway to attenuate but not abolish NFκB as an alternative therapeutic strategy for IBD.

## Methods

### Cell culture and expression constructs

HCT116 and HEK293T (ATCC) cells were grown in DMEM and 10% fetal bovine serum (FBS). All cell lines were purchased from ATCC and distributed from cell culture core of the Department of Cell Biology at Baylor College of Medicine. The cell culture core has specialized staff to test mycoplasma contamination monthly or every other month. pCDNA5/FRT/TO-REGγ, pGEX-4T-1-IκBɛ, pCDNA3.1-IκBɛ, pCMVtag2B-IκBɛ and pCDNA3-GFP-REGγ were previously constructed. NF-κB-Luc reporter plasmid was a kind gift from Dr Jianhua Yang, Baylor College of Medicine.

### Experimental mice

REGγ ^−/−^ mice with C57BL/6 genetic background were kindly provided by Dr John J. Monaco at University of Cincinnati^23^ and backcrossed at our facility for more than 10 generations. C57BL/6 IκBɛ^−/−^ mice were previously generated^52^. All experiments were conducted with 7–10-weeks-old male mice housed under specific pathogen free (SPF) conditions and handled according to the ethical and scientific standards by the Animal Center at the institute (Minhang Laboratory Animal Center East China Normal University). Details of animal studies were described in figure legends and following sections according to the ARRIVE guidelines^53^.

### Immunoprecipitation and western blotting

Cells were transfected with constructs or treated as explained in the figures. Cells were then scraped into ice-cold PBS and lysed with lysis buffer containing 50 mM Tris-HCl, pH7.5, 1 mM EDTA, 1% NonidetP-40, 150 mM NaCl, 10% glycerol and protease inhibitors for 30 min on ice. Then centrifuge for 10 min at 12,000 r.p.m.Specific proteins were immuneprecipitated, followed by three washes with wash buffer (50 mM Tris-HCl, pH 7.5, 1 mM EDTA, 0.1% Nonidet P-40, 150 mM NaCl, 10% glycerol and protease inhibitors). The pellet was then resuspended in SDS sample buffer and resolved in 4–15% gradient SDS gels. Separated proteins were transferred to nitrocellulose membranes and immunoblotted with primary antibodies. β-actin (A2228, Sigma; the dilution ratio is 1:5,000) was used as a loading control. P-p65 antibody (#3033,93H1; the dilution ratio is 1:2,000) was purchased from Cell Signaling Technology. Blots were then incubated with horseradish peroxidase-conjugated secondary antibody (Goat Anti-Mouse IgG (H+L), 115-035-166, Jackson ImmunoResearch; Goat Anti-Rabbit IgG (H+L) 111-035-144; the dilution ratio is 1:5,000) and visualized by chemiluminescence. Images have been cropped for presentation. Full size images are presented in Supplementary Figs 8–12.

### GST pulldown assays

GST-IκBs or GST-IκBɛΔN60 protein was purified using glutathione sepharose affinity chromatography (Bio-Rad). REGγ protein was expressed in *Escherichia coli* from pPAL7-REGγ vector and was purified by Profinity eXact affinity chromatography with fast protein liquid chromatography(FPLC) system. The Profinity eXact tag was removed enzymatically from REGγ in Buffer P2 (68.4 mM Na_2_HPO_4_, 31.6 mM Na_2_HPO_4_, 100 mM NaF, 1 mM DTT and 0.1 mM EDTA) after 16 h incubation. Direct physical interactions between IκBs or IκBɛ ΔN60 and REGγ were assessed by incubating equal amounts of GST- IkBs or GST-IκBɛΔN60 proteins with REGγ in binding buffer (50 mM Tris-HCl (pH 7.5), 200 mM NaCl, 10% glycerol, 1% NP-40, 1 mM DTT plus protease inhibitor cocktail). After extensive washes, bound proteins were examined by western blotting.

### *In vitro* proteolytic analysis

Recombinant REGγ protein used herein was purified as described above. The substrate proteins were generated by *in vitro* translation. The proteolytic assays were performed by incubating substrate, 20S proteasome (Boston Biochem) and REGγ heptamers for 1 h in 50 ml reaction volume at 30 °C with proper controls. An aliquot of the reaction was analysed by western blotting.

### Immunohistochemistry

For H&E staining and IHC, mouse colons were fixed overnight in 2% paraformaldehyde, transferred into gradient ethanol, rolled, processed and embedded into paraffin. Anti-IκBɛ antibody (sc-7155, Santa Cruz; the dilution ratio is 1:300), anti-Ly6G antibody (ab25377, abcam; the dilution ratio is 1:2,000), anti-CD11c antibody (ab33483, abcam; the dilution ratio is 1:1,000) and anti-F4/80 antibody (ab6640, abcam; the dilution ratio is 1:1,000) were purchased from indicated companies. Sections were cut at 4 μm.

### Luciferase assays

After transfection of indicated plasmids and/or TNF/LPS treatment, the cells were collected and washed with cold PBS once. The cells were then lysed in cell lysis buffer (Promega). Following one freezing and thawing cycle, the whole-cell lysates were centrifuged in cold room (4 °C) at 12,000 r.p.m. for 10 min. Supernatant was collected in a fresh tube and 20 μl of it was added to equal amount of luciferase assay substrate. Luminescence was detected as relative light units using a LUMIstar OPTIMA (BMG Labtech) reader. Each assay was repeated for three times. Fold change values were represented as mean of the three experiments.

### Electrophoretic mobility shift assay

Nuclear extracts were generated from colon epithelial cells of mice with various genotypes using a nuclear and cytoplasmic extraction kit as previous mentioned^19^. Equal amounts of nuclear extracts (2.5 μg) were preincubated with antibodies specific for RelA (sc-372, Santa Cruz) or controls at room temperature for 30 min. Following the preincubation with antibodies, Alexa fluor 680-labelled consensus NFκB probes were added and incubated at room temperature for an additional 30 min. The resulting DNA/protein/Ab complexes were resolved by electrophoresis on a 6% nondenaturing polyacrylamide gel and recorded by Odyssey (Leica).

### ChIP assay

Nuclear proteins were crosslinked to genomic DNA by adding 1% formaldehyde for 10 min.Crosslinking was stopped by adding 0.125 M glycine. Then the cells were collected, resuspended in lysis buffer (1% SDS, 10 mM EDTA, protease inhibitors and 50 mM Tris-HCl (pH 8.1)) and the lysates were sonicated to result in DNA fragments of 200–1,000 bp in length. DNA fragments were extracted from chromatin IP by adding 4 μl anti-p65 Ab (sc-372, Santa Cruz) with phenol-chloroform. PCR amplification of the genomic DNA was performed with specific primers. Human IL-8 primers: forward, 5′-GGGCCATCAGTTGCAAATC-3′ and reverse, 5′-TTCCTTCCGGTGGTTTCTTC-3′. PCR product was separated by 2% agarose gel electrophoresis and visualized by UV.

### Induction of DSS-induced colitis and colorectal cancer

Acute colitis was induced with 2% (w/v) DSS (molecular mass 36–40 kDa; MP Biomedicals) for 7 days. Mice were randomly grouped with different genotypes in separate cages. For the colitis-associated colon cancer model, mice were given i.p. injection with 10 mg kg^−1^ AOM (Sigma). Seven days later, 2% DSS was given in drinking water over 7 days, followed by normal water until mice were killed^30^.

### Clinical scoring of colitis and histopathological analysis

The DAI is the combination of weight loss, stool consistency and rectal bleeding, leading to a maximum DAI of 12. Briefly, weight loss scores were determined as follows: 0=none; 1=1–5% loss; 2=5–10% loss; 3=10–15% loss; and 4=15–20% loss. Stool consistency scores were determined as follows: 0=normal; 1=semi-normal; 2=loose stool; 3=loose stool that adhered to the anus; and 4=liquid stools that adhered to the anus. Rectal bleeding scores were determined as follows: 0=normal; 1=semi-normal; 2=positive hemoccult; 3=blood traces in stool visible; and 4=gross rectal bleeding. Distal colon sections were stained with H&E. The degree of colonic injury was coded and assessed blindly by three individuals based on a scale that grades the extent of inflammatory infiltration (0–5), crypt damage (0–4) and ulceration (0–3). The inflammatory infiltration score was defined as follows: 0=no infiltrate; 1=occasional cell limited to submucosa; 2=significant presence of inflammatory cells in submucosa, limited to focal areas; 3=infiltrate present in both submucosa and lamina propria, limited to focal areas; 4=large amount of infiltrate in submucosa, lamina propria and surrounding blood vessels, covering large areas of mucosa; and 5=transmural inflammation. The crypt damage score was defined as follows: 0=none; 1=some crypt damage, spaces between crypts; 2=larger spaces between crypts, loss of goblet cells, some shortening of crypts; 3=large areas without crypts, surrounded by normal crypts; and 4=no crypts. The ulceration score was defined as follows: 0=none; 1=small, focal ulcers; 2=frequent small ulcers; and 3=large areas lacking surface epithelium.

### Bioinformatics analysis

Microarray datasets were analysed by arrayQualityMetrics, affyQCReport and affy packages from bioconductor http://www.bioconductor.org/ with R http://www.r-project.org/. First, raw data were downloaded from NCBI Gene Expression Omnibus (GEO, http://www.ncbi.nlm.nih.gov/geo) database (ID:GSE10616). Second, 53 samples were chosen from the data sets and grouped into four classes, namely healthy control group (*n*=11), CD-only patients (*n*=14), ileo-colonic CD patients (*n*=18) and UC patients (*n*=10). Collected data were normalized by robust multi-array average expression measure depending on affy packages in R. The log2 ratios of gene expression values were calculated based on the normalized data. To detect whether *REGγ* gene is differentially expressed among these four groups, we carried out the following statistical analysis. With all data passing the Shapiro–Wilk test (known as W-test) and Bartlett's test to ensure equality of data variation, one-way analysis of variance method was used to analyse the means of these four groups. Holm method was used to adjust *P* value in paired *t*-test. All statistical analysis was performed in R.

### Colon organ explant culture and measurement of cytokines

The distal section of colon was excised and cut into 1 cm^2^ sections. Tissues were washed in PBS containing penicillin and streptomycin, and the weight of each section was recorded. The colon section was placed in complete RPMI medium 1640 (10% FBS, 1% penicillin and streptomycin) and cultured at 37 °C for 24 h. The supernatants were harvested and cytokines were measured by ELISA from BioPlex Multiplex (Bio-Rad) according to the manufacturer's instructions. CXCL-5 was measured using an ELISA Kit (abcam).

### Expression profiling

Total RNA was isolated from cultured cells, isolated colon epithelial cells or mouse colon tissues using TRIZOL (Takara), following the manufacturer's protocol. Briefly, 0.5–2 ug of total RNA was reverse-transcribed to cDNA. For quantitative RT–PCR analysis, the reverse-transcribed cDNA was subjected to RT–PCR using a master-mix with SYBR-green (TOYOBO) and the Mx3005P quantitative RT–PCR system (Agilent). Each experiment was performed in duplicates and was repeated at least three times. For RT–PCR of mouse cells/tissues, results were average from more than six mice. The related primers are shown in Supplementary Table 3.

### Isolation of epithelial cells

Colons were dissected, washed with cold PBS, and cut into small pieces. The minced tissues were incubated with Hanks-balanced salt solution (HBSS) supplemented with 1 mM DTT, 5 mM EDTA and antibiotics at 37 °C for 30 min with gentle shaking. This process was repeated twice to collect more epithelial cells.

### Isolation of lamina propria mononuclear cells

After removing epithelial layer as above, the remaining sections were incubated at 37 °C in HBSS containing 0.05% Collagenase D (Roche), 0.05% DNase I (Sigma) and 0.3% Dispase II (Roche) for 30 min with gentle shaking. After digestion, the supernatant was passed through a 70 μm cell strainer (BD Falcon). The filtrate was centrifuged and the pellet was resuspended in 40% Percoll (GE Healthcare). Then overlay the cell suspension on top 80% Percoll and Centrifuge the Percoll gradient for 20 min at 1,000*g*. Lamina propria mononuclear cells were collected from the 40/80% interface.

### Generation of bone marrow chimaeric mice

Recipient *WT* or *REGγ*^−/−^ mice were irradiated with 9 Grays of X-rays, and bone marrow cells (isolated from femur and tibia) were injected i.v. via the tail (1 × 10^7^ per mouse). Four chimera groups were generated: *WT*–*WT*; *REGγ*^−/−^ –*WT*; *WT*–*REGγ*^−/−^; and *REGγ*^−/−^–*REGγ*^−/−^. Mice were housed for 2 months before induction of DSS colitis.

### Flow cytometric analysis

Colonic lamina propria mononuclear cells, mesenteric lymph node (MLN) and spleen cells were stained for surface makers CD4-APC (RM4-5), B220-PerCP-Cy5.5 (RA3-6B2), CD11b-APC (M1/70), CD11c-PE (N418) and Gr-1-PerCP-Cy5.5 (RB6-8C5) (eBioscience). Stained cells were analysed by BD FACS LSRII and further analyses were performed with FlowJo software.

### Antibody arrays

The cell lysates from *REGγ*^+/+^ and *REGγ*^−/−^ MEF cells were carried out a high-throughput proteomic screen of potential *REGγ* targets using antibody arrays (FullMoon BioSystems). The Full Moon arrays contain antibodies against nearly 1,300 phospho and total proteins, which involves in more than 30 different regulatory pathways.

### Analysis of human colitis samples

Human sample study was approved by the independent ethics committee at the Fifth hospital of Shanghai, Fudan University. All UC or control (routine analysis) samples were from colonoscopy. All clinical samples were devoid of personal information. The sections were counterstained with haematoxylin and the staining intensity was evaluated on a scale of 0–3, and was rated as negative (–), weak staining (+), moderate/strong staining (++) and very strong staining (+++).

### BMDM and BMDN

Bone marrow-derived macrophages were flushed out from the femur with ice-cold HBSS. The cell were seeded in RPMI1640 supplemented with 10% of heat-inactivated FBS and 100 ng ml^−1^ mouse colony-stimulating factor (Sigma-Aldrich). After 3 days, non-adherent cells were removed by washing with HBSS and the medium was subsequently replaced daily until cells were harvested. After 6 days, adherent cells were scraped down and cultured in new plates for indicated stimulation. For bone marrow-derived neutrophil, erythrocyte lysed bone marrows were resuspended by 45% gradient percoll, 4 °C, 1500, g for 30 min by using 81%, 62% and 45% gradient percoll to obtain mature neutrophil, then treated with LPS (100 ng ml^−1^) or TNFα (20 ng ml^−1^).

### Statistical analysis

Prism software (GraphPad Software) was used for statistical analyses. Values are shown as mean±s.e.m. Statistical significance between two samples was determined with two-tailed Student's *t*-test.

## Additional information

**How to cite this article:** Xu, J. *et al.* The REGγ-proteasome forms a regulatory circuit with IκBɛ and NFκB in experimental colitis. *Nat. Commun.* 7:10761 doi: 10.1038/ncomms10761 (2016).

## Supplementary Material

Supplementary Information Supplementary Figures 1-12 and Supplementary Tables 1-3
