# Supplementary material for: The REGγ-proteasome forms a regulatory circuit with IκBɛ and NFκB in experimental colitis
Source: Nat Commun. 2016 Feb 22;7:10761. doi: 10.1038/ncomms10761 (PMC4764899; doi:10.1038/ncomms10761)
Supplement: Supplementary Information — Supplementary Figures 1-12 and Supplementary Tables 1-3 [file ncomms10761-s1.pdf]

Supplementary figures

Supplementary Figure 1

A

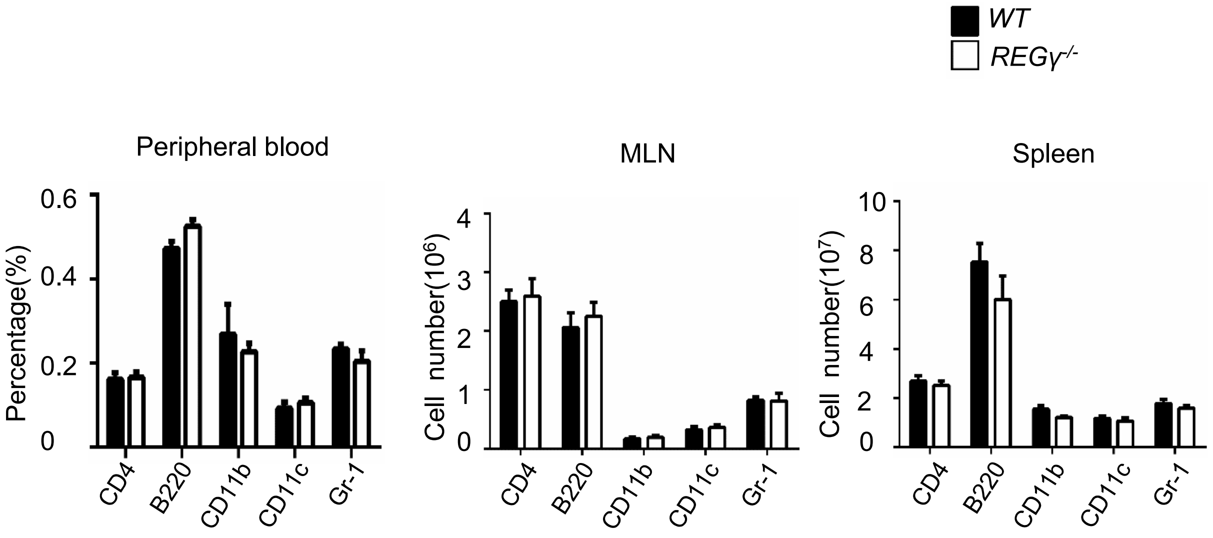

Supplementary Figure 1

No baseline value differences in immune cells from Naïve mice

Peripheral blood, MLN and spleen were collected from Naïve mice. Peripheral blood cells, MLN cells, and splenocytes were analyzed by flow cytometry after staining with antibodies for CD4,B220, CD11b, CD11c, and Gr-1. n=6 per group. Data represent means ± SEM(t-test) .

Supplementary Figure 2

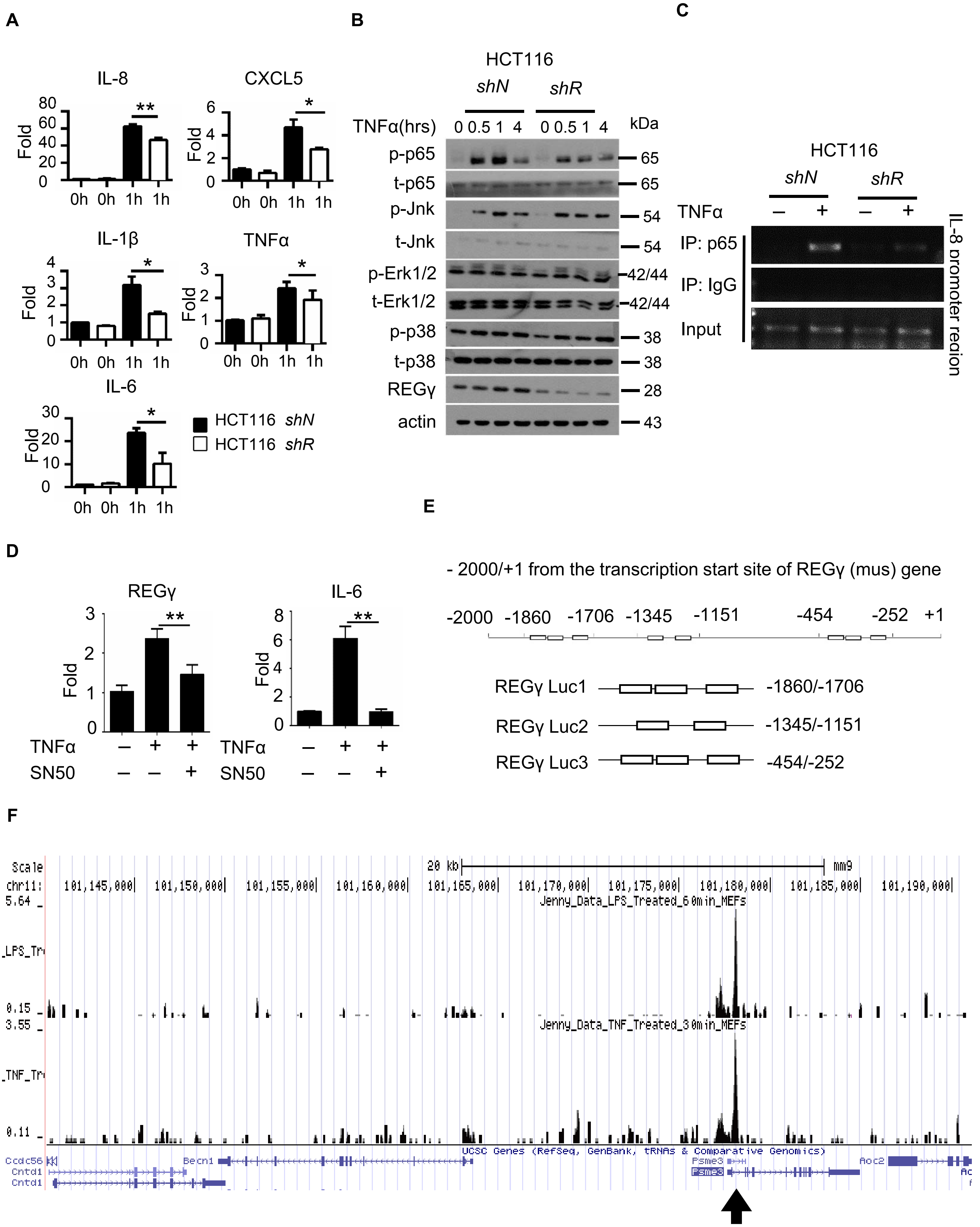

Supplementary Figure 2

Mutual regulation between REGγ and NFκB.

(A) Total RNA was extracted from colon cancer cell line HCT116 stably knocked down REGγ (shR) and control (shN) with or without TNFα stimulation. Expression of NFκB downstream genes were analyzed by RT-PCR.

Data represent means ± SEM from three independent experiments; *p < 0.05; **p < 0.01(t-test).

(B) Alteration of NFκB and MAPK pathways was estimated in HCT116 shN and shR cells.

(C) ChIP assays of p65 recruitment to IL-8 promoter were performed in HCT116 shN and HCT116 shR cells treated with TNFα for one hour. Chromatin was immunoprecipitated with anti-p65 Ab. Ten percent of the precipitated chromatin (input) was assayed to ensure equal loading.

(D) REG γ transcripts were measured before and after 6h TNF treatment with or without pretreatment of NFκB inhibitor in colon ex vivo culture. n=4, each group, Data represent means ± SEM from three independent experiments; *p < 0.05; **p < 0.01(t-test).

(E) Potential NFκB binding sequences on REGγ promoter was analyzed using TFSEARCH software (http://www.cbrc.jp/research/db /TFSEARCH.html) .

(F) ChIP-seq analysis for NFκB/p65 in primary MEFs treated with LPS for 1 hr or TNF for 30 min.

We note a strong peak over the promoter-proximal region of the PSME3/REGγ gene but not neighboring genes.

Supplementary Figure 3

A

	REGγ$^{+/+}$ vs *REGγ*$^{-/-}$	
Name	T-test	Fold change
PKD1/PKCmu(phosphorSer205)	0.00025	0.33217498
c-Jun(phospho-Ser63)	0.00935	0.319666351
Rac1/cdc42(phospho-Ser71)	0.00435	0.302642388
IκB-ε	0.00829	0.27377487
Androgen Receptor(Ab -650)	0.008	0.166621646
Trk A(Ab-496)	0.00116	0.086558242

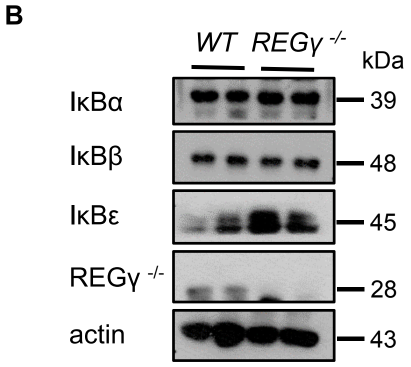

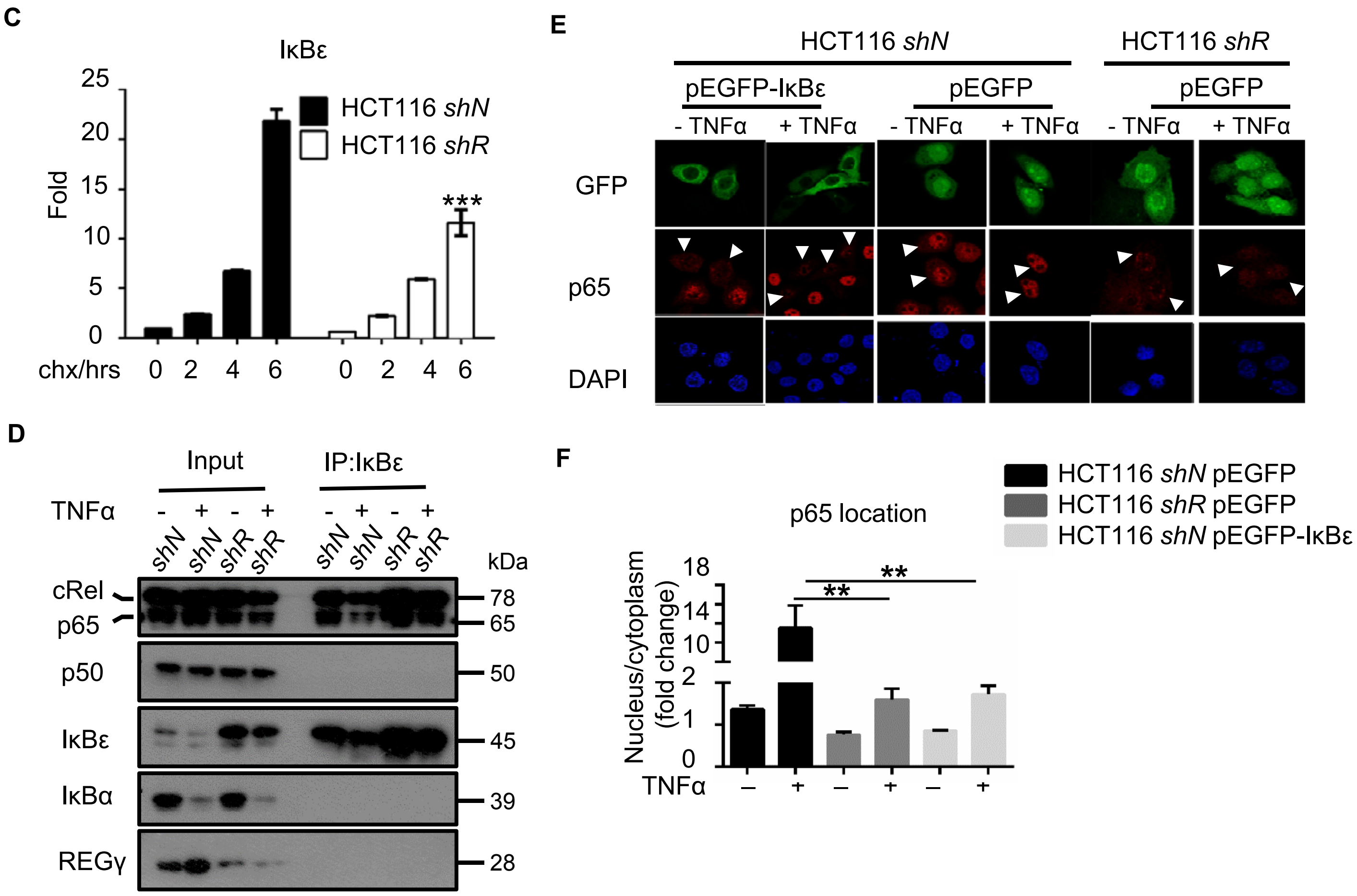

Supplementary Figure 3

REGγ modulating NFκB signaling via regulation of IκBε

(A) High-throughput proteomic screen of antibody arrays (FullMoon Biosystems) revealed differential expression of IκBε in *REGγ*$^{+/+}$ and *REGγ*$^{-/-}$ MEF cell. (B) Expression of IκBs in colon epithelial cells isolated from *WT* and *REGγ-/-* mice following 7 days of DSS treatment. Representative of three successful times.n=7,each group. (C) IκBε mRNA levels were measured in HCT116 *shN* and *shR* cell following CHX treatment. Data represent means ± SEM from three independent experiments; ***p < 0.001(t-test). (D) IκBε protein complexes. HCT116 *shN* and *shR* cell were stimulated with/without TNFα and immunoprecipitated (IP) with IκBε antibody followed by Western blot as indicated. P65 and c-Rel, but not p50, could be immunoprecipitated by IκBε. Moreover, elevated IκBε in *shR* cell contributed to more stable IκBε/p65/c-Rel complex that are resistant to TNFα stimulated degradation. Representative of three experiments.
(E and F) HCT116 *shN/shR* cells transfected with a control vector (pEGFP) or IκBε (pEGFP-IκBε) were stimulated with or without TNFα for 1 hour. Cell were then fixed and labeled for GFP and p65. Quantitative assessment of relative p65 distribution (nucleus/cytoplasm) reflects relative NFκB activities in 3 independent experiments. Data represent means ± SEM , **p < 0.001(t-test).

Supplementary Figure 4

A

B

C

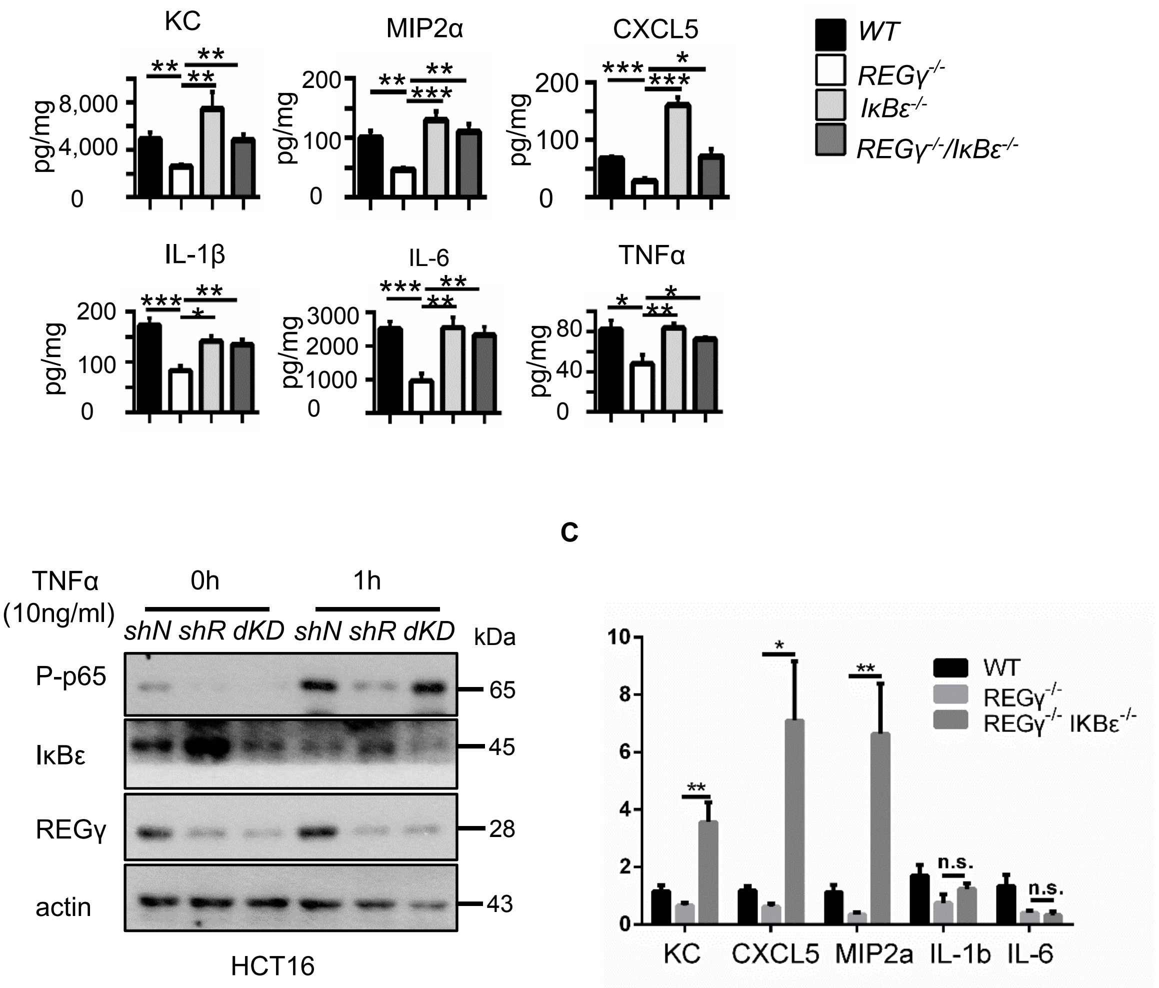

Supplementary Figure 4

IκBε significantly contributes to REGγ-dependent regulation of NFκB responsive genes

(A) Colon levels of cytokines were assessed from supernatants of organ culture by ELISA. n=5,each group. Data represent means±SEM. *p < 0.05; **p < 0.01, ***p < 0.001(t-test).

(B) Compound depletion of IκBε and REGγ restores NFκB activity in HCT116 *dKD* (*REGγ* and *IκBε* stable knock-down) cells. Representative of two experiments.

(C) Colon epithelial cells from *WT, REGγ$^{-/-}$* and *REGγ$^{-/-}$ /IκBε$^{-/-}$* mice were isolated at experimental day 7, total RNA was extracted for analysis of NFkB regulated down-stream genes by real- time RT-PCR; n= 5,each group . Data represent means±SEM. *p < 0.05; **p < 0.01; n.s.=no significance (t-test).

Supplementary Figure 5

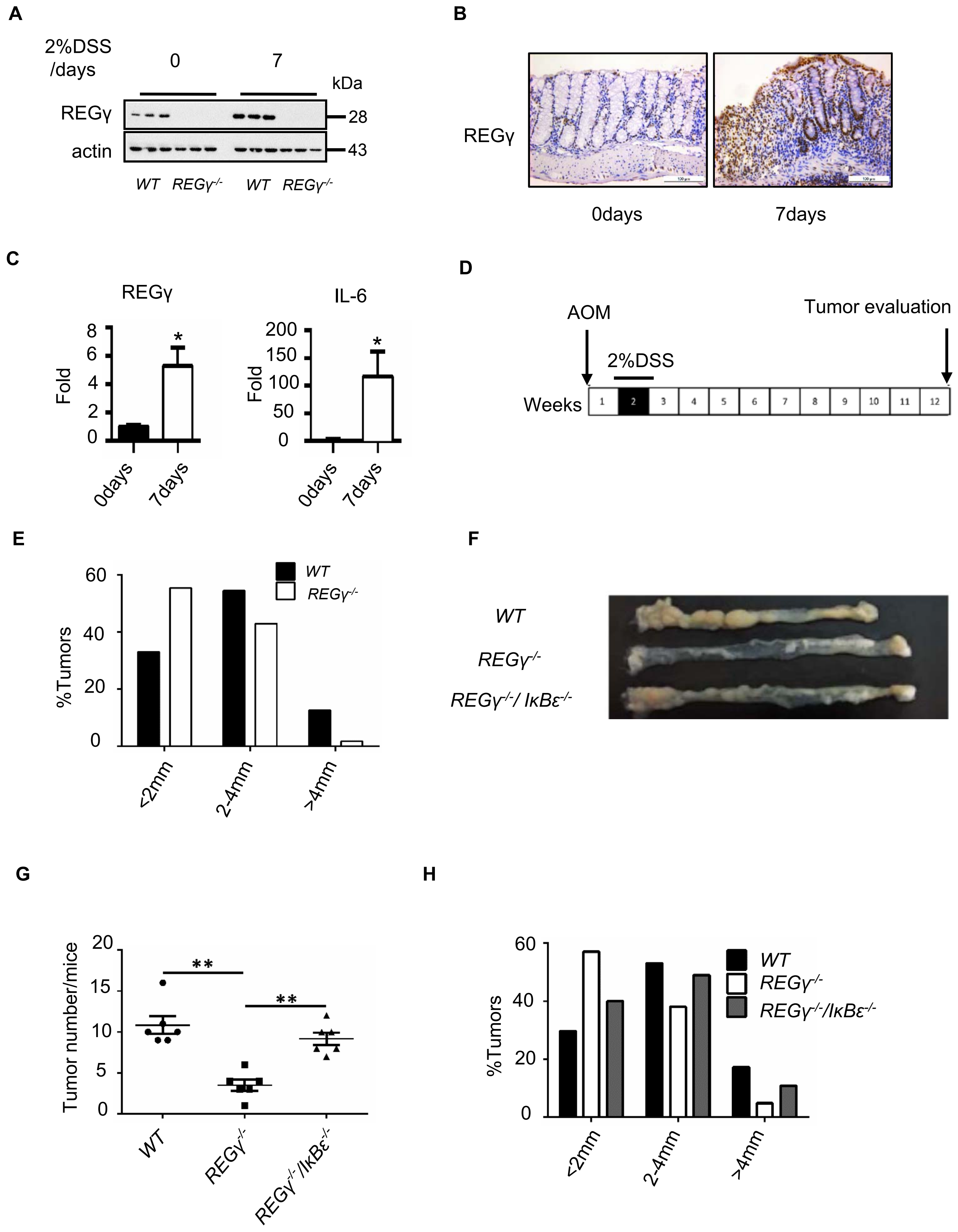

Supplementary Figure 5

Elevated REGγ expression in colitis is associated with colon tumorigenesis in an IκBε dependent manner

(A) REGγ protein level was measured in colon epithelial cells from day 0 or 7 days DSS treated mice by Western blot.

(B) Analysis of REGγ protein level in paraffin-embedded colon sections of control (day 0) or 7 days DSS treated mice by immunohistochemistry. Representative images were shown, and the experiment was successfully repeated four times. Scale bars represent 100 μM.

(C) Expression of REGγ and inflammatory cytokine IL6 were elevated in colon epithelial cells collected from DSS treated mice, n=6,each group. Data shown are representative of three independent experiments and denotes means ± SEM. *p < 0.05.

(D) Schematic diagram of the experimental design for colitis-associated tumorigenesis. Mice were injected with AOM followed by one round of 2% DSS treatment for 7 days.

(E) Diameter of the tumors in *WT* and *REGγ*$^{-/-}$ mice was measured. n =8,each group .

(F) Representative appearances of colon tumors developed in *WT, REGγ*$^{-/-}$*,* and *REGγ/IκBε* double knockout mice after AOM and DSS induction. n =8,each group .

(G) The number of colon tumors in *WT, REGγ*$^{-/-}$*,* and *REGγ/IκBε* double knockout mice was counted after AOM and DSS induction. n= 11,*WT* group; n=10,*REGγ*$^{-/-}$ group; n=9,*REGγ/IκBε* double knockout group. **p < 0.01.

(H) Summary of the tumor size in mice with different genotypes.

Supplementary Figure 6

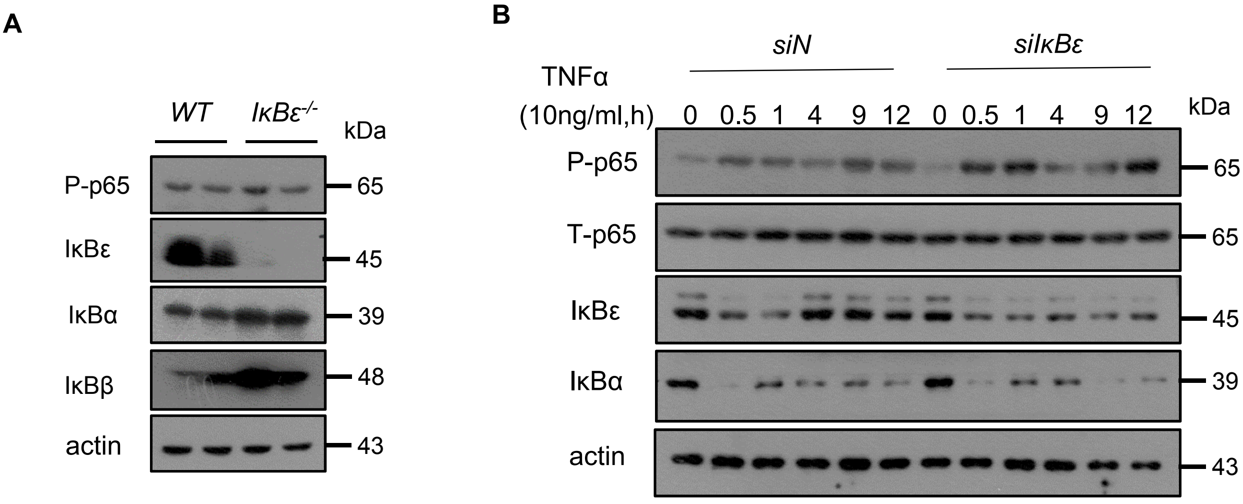

Supplementary Figure 6

Compensational upregulation of other IκBs in *IκBε*$^{-/-}$ colon epithelial cells.

(A) Expression of IκBα/β and p-p65 in colon epithelial cells isolated from *WT* and *REGγ*$^{-/-}$ mice following 7 days of DSS treatment. Each lane represents a sample from an individual mouse. Representative data were from three experiments.

(B) Silenced IκBε with transient siRNA in HCT116 cell to detect p-p65 and IκBα.

Supplementary Figure 7

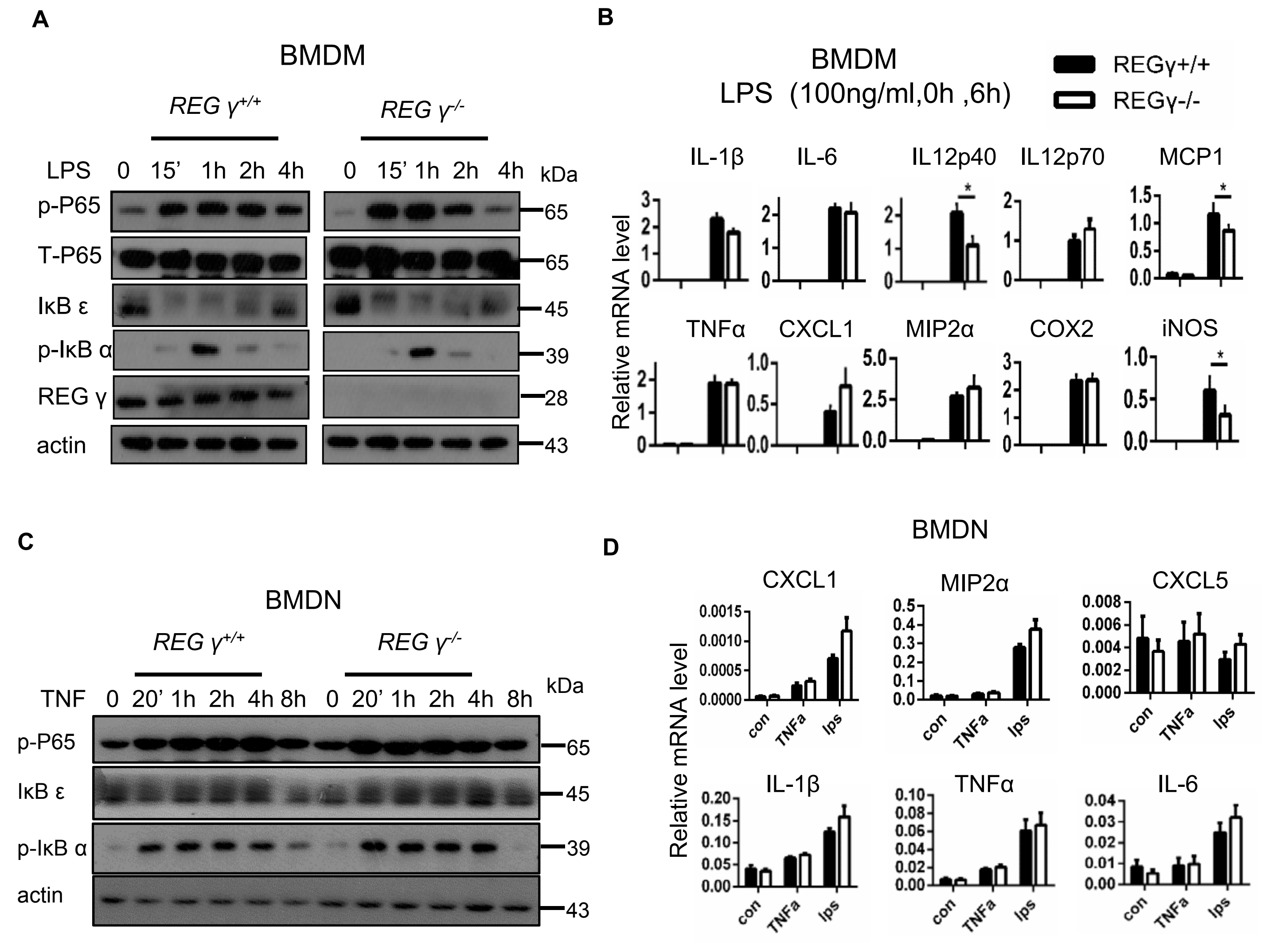

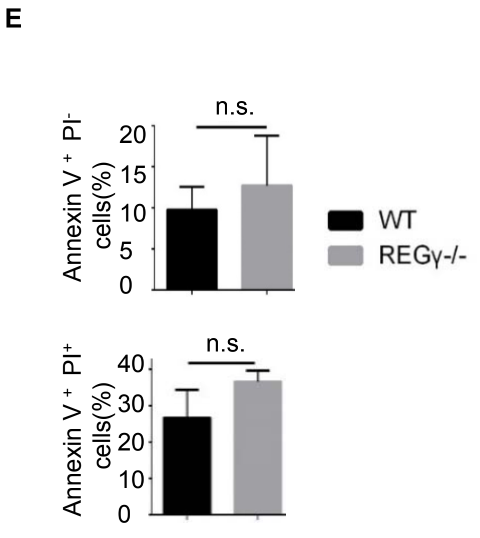

Supplementary Figure 7

Comparable immune cell responses in vitro and apoptotic responses in DSS-colon between *WT* and *REGγ*$^{-/-}$ mice

(A) BMDM were treated with LPS (100ng per ml) for indicated time and analyzed for NFκB activity by Western blot. Representative images were from three successful repeats. (B) BMDM were treated with LPS(100ng per ml) for 0h, 6h, and analyzed for expression of inflammatory genes by Q-PCR. n=6,each group. Data represent means ± SEM from three independent experiments. *p < 0.05, Student's t-test. (C) BMDN were treated with TNFa (20ng per ml) for indicated time and analyzed for NFκB activity by Western blot. Representative images were from three successful repeats. (D) BMDN were treated with TNFa (20ng per ml) or LPS (100ng per ml) for 6h, and analyzed for expression of inflammatory genes by Q-PCR. n=6,each group. Data represent means ± SEM from three independent experiments. Student's t-test. (E) No significant differences in apoptotoc cell numbers in DSS treated colons between *WT* and *REG γ* $^{-/-}$ mice Colon epithelial cell were collected from *WT* and *REGγ-/-* mice after DSS treatment for 7 days and analyzed by flow cytometry for annexin V and propidium iodide. n=6/each group. Data represent means±SEM. n.s.=none significance.

Supplementary Figure 8

Uncropped gel images for Figure 4

Figure 4 A

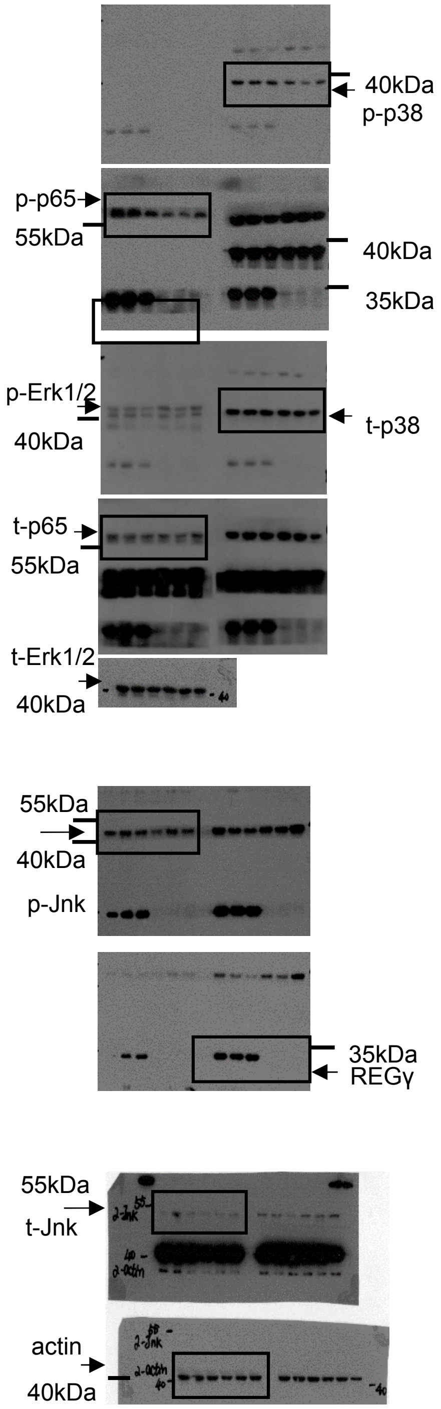

Figure 4 B

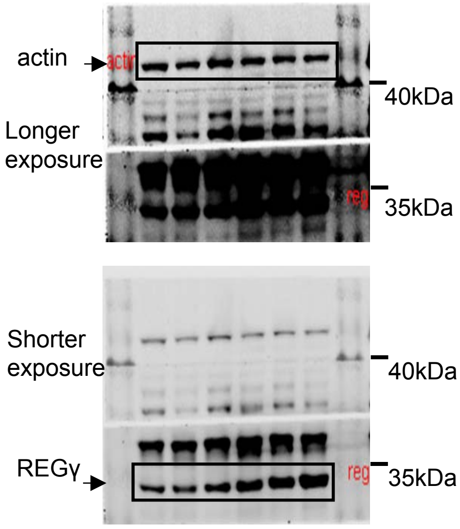

Figure 4 C

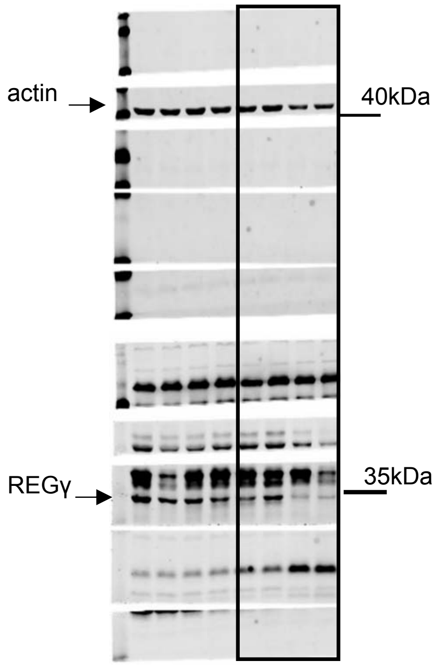

Figure 4 E

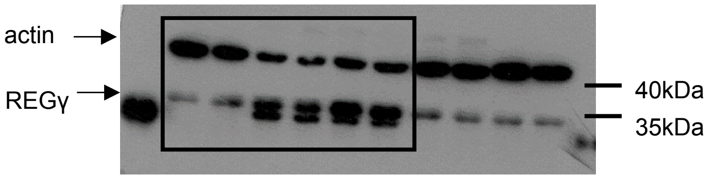

Figure 4 F

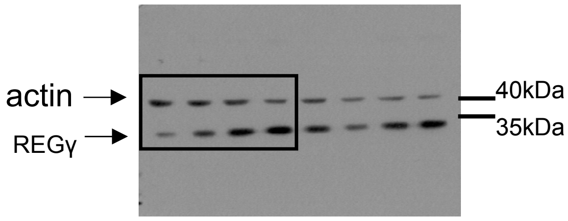

Figure 4 I

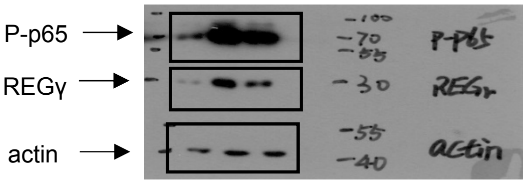

Supplementary Figure 9

Uncropped gel images for Figure 5A-G

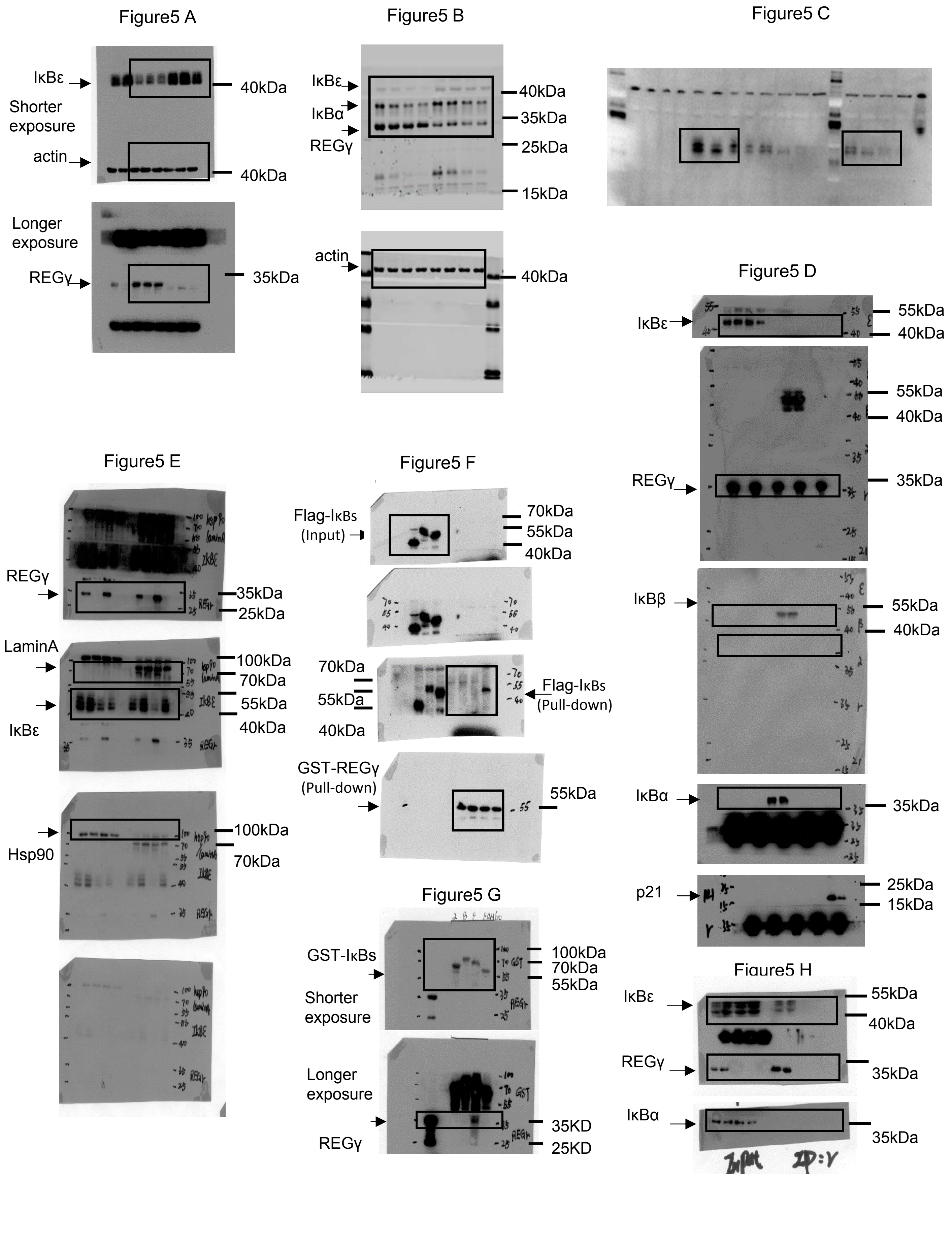

Supplementary Figure 10

Uncropped gel images from Figure 5H and Figure 6

Figure5 I

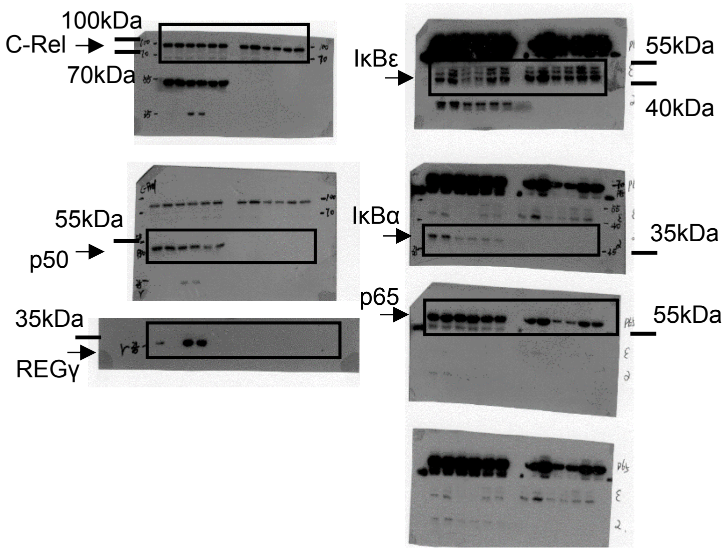

Figure 6 G

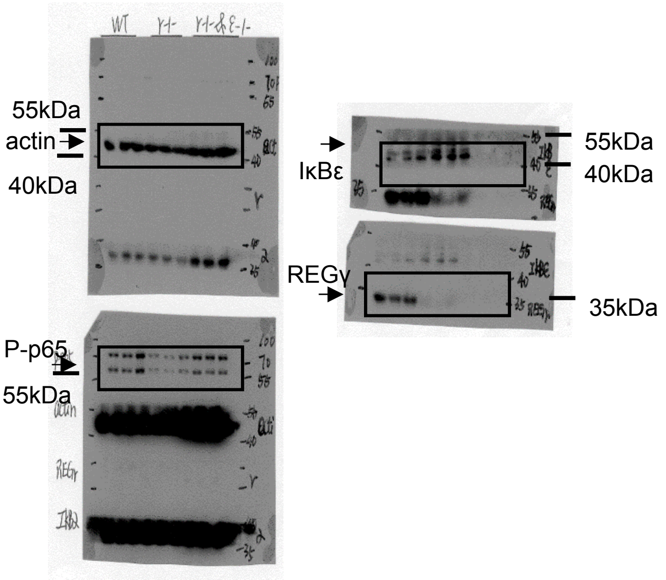

Supplementary Figure 2 B

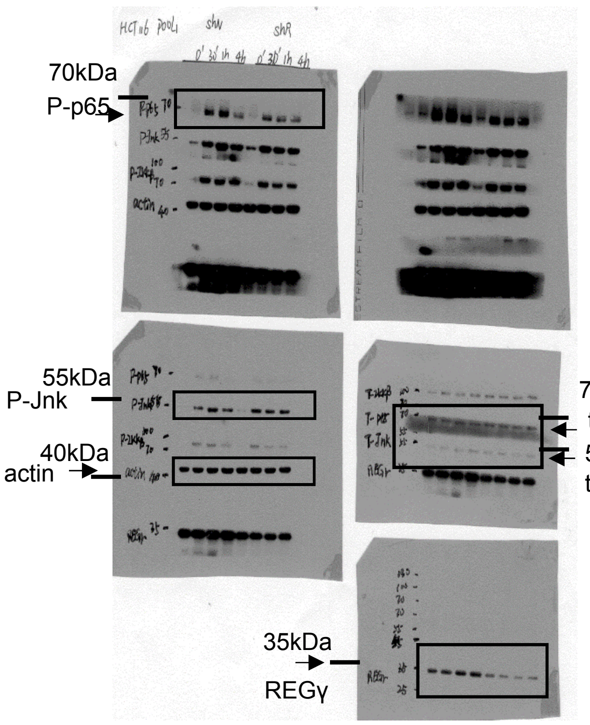

Supplementary Figure 3 B

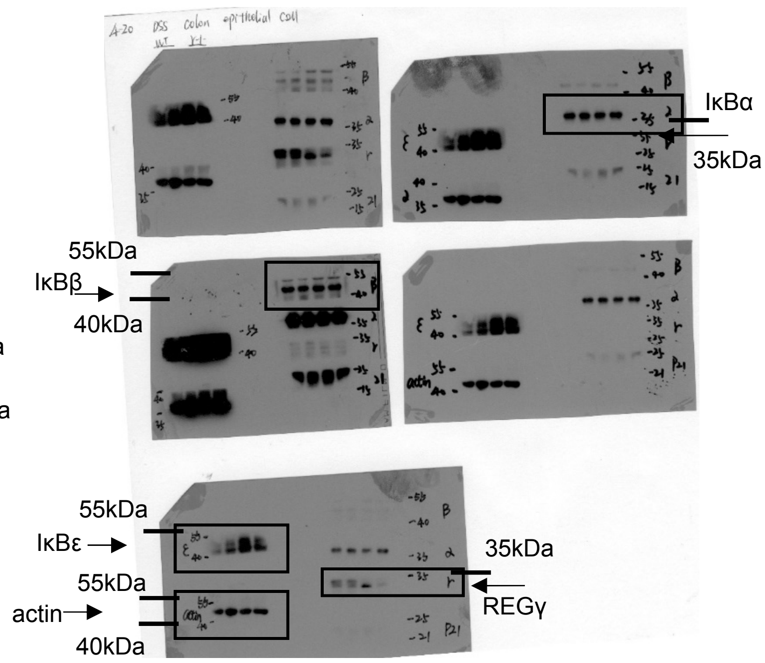

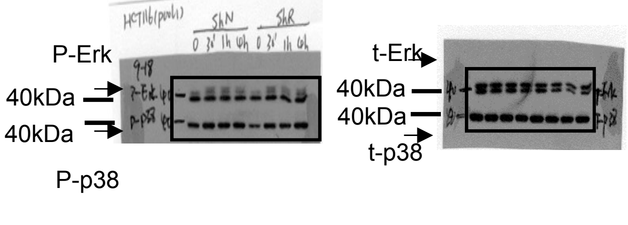

Supplementary Figure 11

Supplementary Figure 3 D

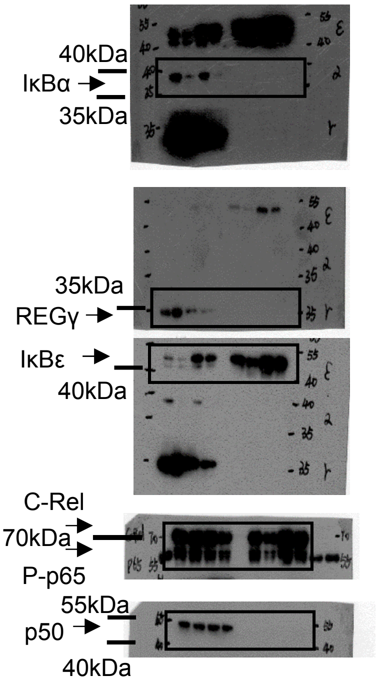

Supplementary Figure 4 B

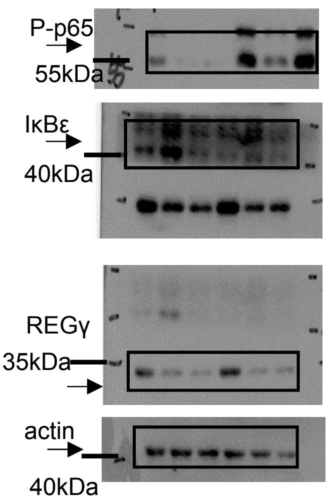

Supplementary Figure 5 A

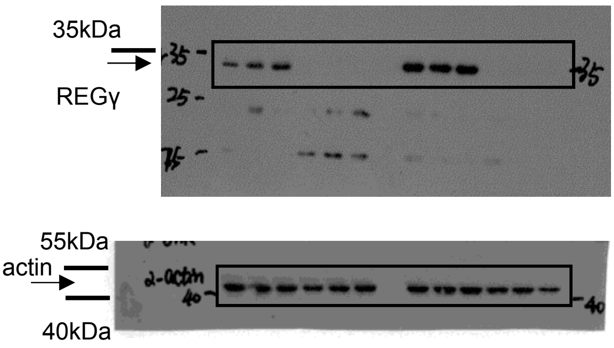

Supplementary Figure 6 A

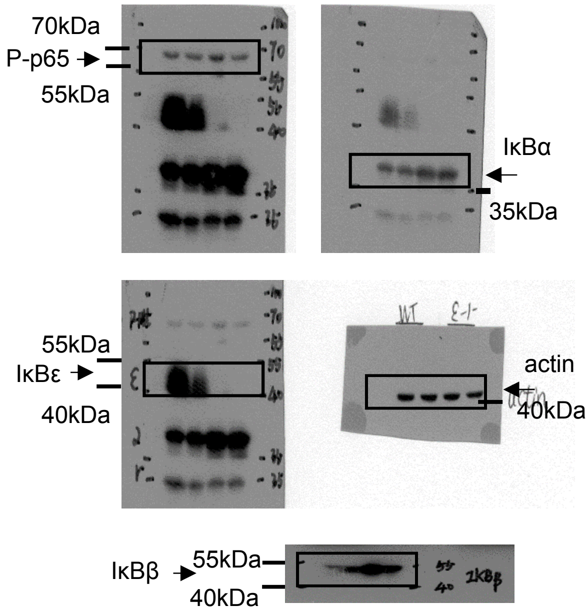

Supplementary Figure 6 B

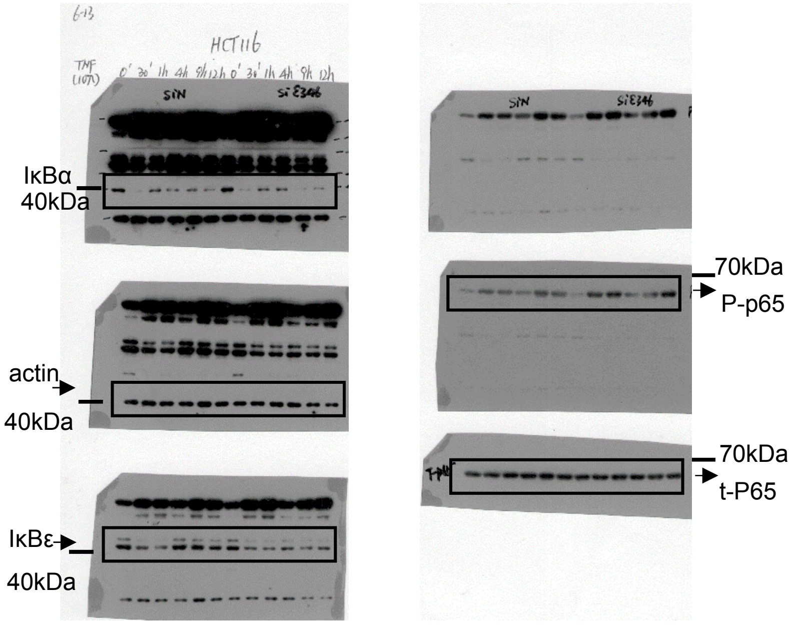

Supplementary Figure 12

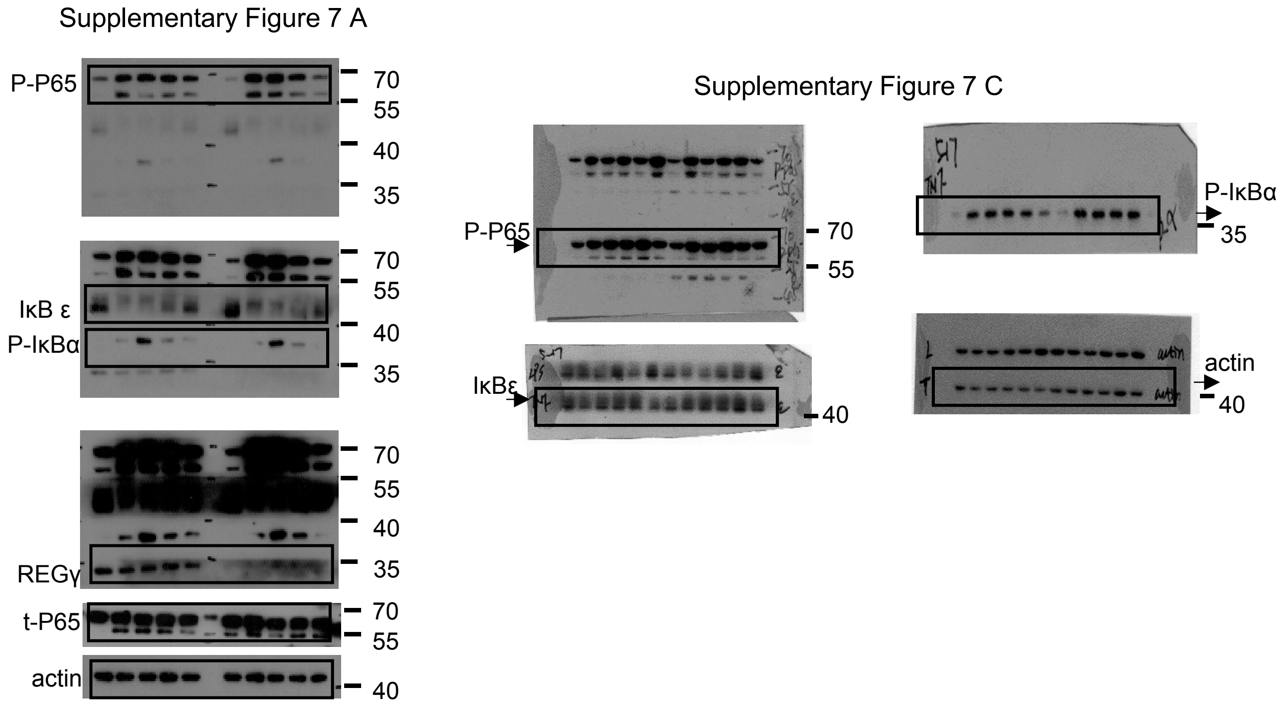

Supplementary Tables

Supplementary Table 1

Proteins differentially regulated in REGγ$^{+/+}$ and REGγ$^{-/-}$ MEF cells. The Full Moon arrays contain antibodies against nearly 1,300 phospho and total proteins, which involves in more than 30 different regulatory pathways.

	**REGγ$^{+/+}$ vs REGγ$^{-/-}$**		**REGγ$^{+/+}$ vs REGγ$^{-/-}$**
**Name**	**Fold change**	**Name**	**Fold change**
BCL-2 (Ab-70)	10.28896725	PAK1/2/3(PhosphoSer141)	1.677577155
Rb (Ab-608)	7.926245366	GluR1 (Phospho-Ser863)	1.668237568
CREB (Phospho-Ser142)	7.795426163	MYPT1(PhosphoThr853)	1.66785366
Vinculin (Ab-821)	3.581243833	MER/SKY(PhosphoTyr749/Tyr681)	1.663813763
PKC pan activation site	3.186660576	ASK1 (Phospho-Ser966)	1.66351219
ACC1 (Phospho-Ser80)	3.058972685	Pyk2 (Ab-881)	1.657414582
c-PLA2 (Phospho-Ser505)	3.00150248	ALK (Phospho-Tyr1507)	1.651515091
Smad3 (Ab-204)	2.778798545	CDK7 (Phospho-Thr170)	1.651493086
ATF-1 (Ab-63)	2.774254867	CASP9 (Ab-125)	1.650001352
Rb (Phospho-Ser795)	2.667937343	c-Jun (Ab-93)	1.640771286
PKCdelta(PhosphoSer645)	2.635777114	MKP-1/2 (Phospho-Ser296)	1.601532637
c-Jun (Phospho-Thr91)	2.298132554	FER (Phospho-Tyr402)	1.594996135
HDAC4 (Phospho-Ser632)	2.269667543	CD227/mucin1 (Phospho-Tyr1243)	1.583746785
Caspase3(PhosphoSer15)	2.123824406	CDC25C (Phospho-Ser216)	1.567054694
IL3R (Phospho-Tyr593)	2.101396707	ACC1 (Phospho-Ser79)	1.544646342
FER (Phospho-Tyr402)	2.093579469	CD32 (FcgammaRIIb) (Ab-292)	1.518937175
GSK3 alpha (Ab-21)	2.082011346	NFkB-p65 (Phospho-Ser536)	1.509447457
p53 (Phospho-Ser46)	2.062507592	SHP-2 (Phospho-Tyr542)	1.489058626
CASP2 (Ab-157)	2.051445315	merlin (Ab-10)	1.483177065
Chk1 (Phospho-Ser317)	2.038564405	RelB (Phospho-Ser552)	1.471011976
P70S6K (Phospho-Ser411)	2.037074234	HDAC8 (Ab-39)	1.469499176
DARPP-32(PhosphoThr34)	1.976117209	PPAR-b (Phospho-Thr1457)	1.466827453
ATPase (Phospho-Ser16)	1.97411589	P38 MAPK (Phospho-Tyr182)	1.455534652
XIAP (Phospho-Ser87)	1.973606985	ITGB4 (Phospho-Tyr1510)	1.442747299
Cateninbeta(PhosphoSer3)	1.972654212	VEGFR2 (Phospho-Tyr1214)	1.438017595
AKT1 (Ab-308)	1.96803916	PAK1 (Phospho-Ser204)	1.433107907
MAP3K7/TAK1 (Ab-439)	1.958481953	Caspase 9 (Ab-196)	1.41905323
HSP 90-beta (Ab-226)	1.810567145	Connexin 43 (Ab-367)	1.419050468
HSP27 (Phospho-Ser78)	1.798735075	NFkB-p65 (Phospho-Ser311)	1.408624928
WASP (Ab-290)	1.732364028	Cyclin E1 (Ab-77)	1.402830933
Dok-1 (Phospho-Tyr362)	1.706018808	p53 (Phospho-Ser392)	1.402106832
MKP-1/2(Phospho-Ser296)	1.697008766	NFkB-p65 (Phospho-Ser276)	1.400258618

	REGγ$^{+/+}$ vs REGγ$^{-/-}$		REGγ$^{+/+}$ vs REGγ$^{-/-}$
Name	Fold change	Name	Fold change
CoagulationFactorIII(PhosphoSer290)	0.708761709	Stathmin 1(Phospho-Ser37)	0.585807389
Rb (Ab-608)	0.706057827	Mst1/Mst2 (Phospho-Thr183)	0.573902117
CD4 (Ab-433)	0.705454713	EGFR (Phospho-Thr678)	0.56397586
FADD (Phospho-Ser194)	0.703367687	c-Jun (Ab-239)	0.550986973
NFkB-p100 (Phospho-Ser872)	0.702728445	BCL-2 (Phospho-Thr56)	0.535331342
PAK1/2/3 (Phospho-Ser141)	0.702558538	PAK2 (Ab-192)	0.525479604
Cyclin D3 (Phospho-Thr283)	0.699571557	Hsp90cochaperoneCdc37(PhosphoSer13)	0.520333639
AKT1 (Phospho-Thr72)	0.699309765	MSK1 (Phospho-Thr581)	0.518629456
COT (Ab-290)	0.696072196	AKT (Phospho-Thr308)	0.516535705
Tuberin/TSC2 (Ab-939)	0.695390025	CDC25A (Ab-75)	0.508631016
FADD (Phospho-Ser194)	0.693457483	RSK1/2/3/4 (Ab-221/227/218/232)	0.492045351
MEF2A (Ab-312)	0.676469453	CDK2 (Phospho-Thr160)	0.491958748
claudin 3 (Phospho-Tyr219)	0.675715492	Synuclein alpha (Ab-133)	0.486865466
P70S6K (Ab-229)	0.666299578	KIT (Phospho-Tyr703)	0.484971849
JunB (Phospho-Ser79)	0.66374614	Shc (Phospho-Tyr349)	0.472671761
RelB (Ab-552)	0.662495301	ATPase (Ab-16)	0.444124652
p21Cip1 (Phospho-Thr145)	0.660524367	KIT (Phospho-Tyr703)	0.440843615
G3BP-1 (Ab-232)	0.660333688	MAPKAPK2 (Phospho-Thr334)	0.440795175
MEF2D (Phospho-Ser444)	0.659388153	Abl1 (Ab-754/735)	0.429181608
ATP1A1/Na+K+ ATPase1 (Ab-23)	0.658697445	EGFR (Phospho-Thr693)	0.395265589
Pim-1 (Ab-309)	0.658260504	AMPK1 (Ab-174)	0.387067112
MARCKS (Phospho-Ser158)	0.656677121	Pyk2 (Phospho-Tyr579)	0.382395096
Raf1 (Phospho-Ser338)	0.656422498	Chk2 (Phospho-Thr68)	0.382244761
eEF2K (Ab-366)	0.654316892	Tau (Ab-205)	0.367804061
NFkB-p105/p50 (Ab-932)	0.638685407	Trk A (Phospho-Tyr701)	0.354871313
ATP-Citrate Lyase (Ab-454)	0.638592776	Abl1 (Ab-204)	0.354572601
VASP (Ab-157)	0.63813815	p53 (Ab-376)	0.35227797
ERK3 (Phospho-Ser189)	0.632898703	CASP8 (Ab-347)	0.348369613
BAD (Ab-112)	0.632325906	Tau (Ab-205)	0.347269551
Catenin beta (Phospho-Tyr654)	0.630975201	PKD1/PKC mu (Phospho-Ser205)	0.33217498
4E-BP1 (Phospho-Thr45)	0.630579786	c-Jun (Phospho-Ser63)	0.319666351
CDK1/CDC2 (Ab-14)	0.628359686	Rac1/cdc42 (Phospho-Ser71)	0.302642388
MEF2D (Phospho-Ser444)	0.627745938	IκBε	0.27377487
4E-BP1 (Ab-65)	0.617150926	Androgen Receptor (Ab-650)	0.166621646
NFkB-p65 (Ab-281)	0.61696929	Trk A (Ab-496)	0.086558242
PLC-beta (Phospho-Ser1105)	0.609756074		

Supplementary table2

IκBs N-terminal sequence alignment

```
IKBbeta      -------------------MAGVACL---------------------------------G
IKBalpha     MFQAA------------------------ERPQEWAMEGPRDGLKK--------------
IKBepsino    MSEARKGPDEAEESQYDSGIESLRSLRSLPESTSAPASGPSDGSPQPCTHPPGPVKEPQE
```

Supplementary Table 3

Sequences of primers used for Q-PCR

Gene	Forward (5'→3')	Reverse (5'→3')
IL-8(homo)	GCATAAAGACATACTCCAAACC	AAAACTTCTCCACAACCCTC
IL-6(homo)	AATTCGGTACATCCTCGACGG	TTGGAAGGTTCAGGTTGTTTTCT
IL-1β(homo)	AGCTACGAATCTCCGACCAC	CGTTATCCCATGTGTCGAAGAA
CXCL5(homo)	AGCTGCGTTGCGTTTGTTTAC	TGGCGAACACTTGCAGATTAC
TNFα(homo)	CCTCTCTCTAATCAGCCCTCTG	GAGGACCTGGGAGTAGATGAG
KC(mus)	ACTGCACCCAAACCGAAGTC	TGGGGACACCTTTTAGCATCTT
MIP2α(mus)	ATCCAGAGCTTGAGTGTGACGC	AAGGCAAACTTTTTGACCGCC
MIP2β(mus)	CATAGCCACT CTCAAGGATG	AGAATGCAGGTCCTTCATCATG
CXCL5(mus)	GTTCCATCTCGCCATTCATGC	GCGGCTATGACTGAGGAAGG
IL-1β(mus)	GAAATGCCACCTTTTGACAGTG	TGGATGCTCTCATCAGGACAG
IL-6(mus)	CTGCAAGAGACTTCCATCCAG	AGTGGTATAGACAGGTCTGTTGG
TNFα(mus)	CCTGTAGCCCACGTCGTAG	GGGAGTAGACAAGGTACAACCC
CyclinD1(mus)	GCGTACCCTGACACCAATCTC	CTCCTCTTCGCACTTCTGCTC
Cox2(mus)	CACCCTGACATAGACAGTGAAAG	CTGGGTCACGTTGGATGAGG
Survivin(mus)	TGGCAGCTGTACCTCAAGAA	AGCTGCTCAATTGACTGACG
